# Nanoparticle-mediated dual delivery of MHC class I and II antigens enhances T cell immunity and anti-tumor potency

**DOI:** 10.1016/j.biomaterials.2026.124128

**Published:** 2026-03-14

**Authors:** Enya Li, Nina Butkovich, Jo A. Tucker, Edward L. Nelson, Szu-Wen Wang

**Affiliations:** a Department of Chemical and Biomolecular Engineering, University of California, Irvine, CA 92697, USA; b Department of Medicine, University of California, Irvine, CA 92697, USA; c Chao Family Comprehensive Cancer Center, University of California, Irvine, CA 92697, USA; d Department of Biomedical Engineering, University of California, Irvine, CA 92697, USA; e Institute for Immunology, University of California, Irvine, CA 92697, USA

**Keywords:** Nanoparticles, Cancer vaccines, MHC class I, MHC class II, Antigens, T cells

## Abstract

Nanoparticle (NP)-based cancer vaccine therapies conventionally incorporate tumor-associated antigens or neoantigens that are major histocompatibility complex (MHC) class I-restricted, an approach that educates cytotoxic CD8^+^ T cells to respond to tumors. Helper CD4^+^ T cells can support CD8^+^ T cell-mediated responses and are activated by MHC class II-presented peptides. However, strategies to deliver MHC class I and II antigens using NPs as vehicles have not been systematically examined for anti-cancer immunity. In melanoma and colon carcinoma murine models, we evaluated the effects of transporting MHC class I and class II antigens using different NP-based approaches, with the NPs being within the optimal size range for uptake by antigen-presenting dendritic cells. We found that co-delivering MHC class I and II peptides on dual-antigen NPs increased proliferation of cytotoxic and helper T cells relative to single-antigen NPs alone (i.e., either MHC class I or II peptide on a NP) and to mixtures of the single-antigen NPs. For both tumor models, dual-antigen NPs also elicited higher antigen-specific Th1 responses, including up to 8-fold higher interferon (IFN)-γ secretion. Significantly, immunization with the dual-antigen NPs prolonged survival, with 40% of melanoma- and 71% of colon carcinoma-bearing mice surviving, compared to 0% and 13%, respectively, of those treated with component- and dose-equivalent mixtures of single-antigen NPs. This highlights the importance of the antigen delivery strategy and locale with respect to the NP, with the simultaneous co-delivery of both MHC class I and II antigens on the same NP being critical for anti-tumor potency.

## 1. Introduction

Immunotherapies such as cancer vaccines often aim to modulate the interplay between dendritic cells (DCs) and T cells to promote the activation of antigen-specific, anti-tumor immunity [1–3]. DCs have the unique property of cross-presentation; that is, the ability to present antigen to both CD8^+^ and CD4^+^ T cells on major histocompatibility complex (MHC) class I and MHC class II, respectively. Nanomaterials provide flexibility in design to enable tuning of the resulting immune response [4]. The nanoparticles (NPs) used in this work are preferentially taken up by DCs, and their use in the delivery of tumor-associated antigens (TAAs) has been shown to enhance anti-tumor efficacy relative to TAAs alone (no NPs) [4–6].

Vaccine strategies may focus on activating CD8^+^ T cells alone or both CD8^+^ and CD4^+^ T cells [7–11]. However, the impact of the spatial organization of MHC class I and MHC class II peptides, specifically whether they should be co-delivered on the same NP or on separate NPs, represents an important yet unexamined parameter in NP-based cancer vaccine design. In this study, we examined different configuration strategies to deliver MHC class I and MHC class II antigen epitopes using NPs, with the goal of improving antigen-specific immune responses and increasing survival.

**Fig. 1.**
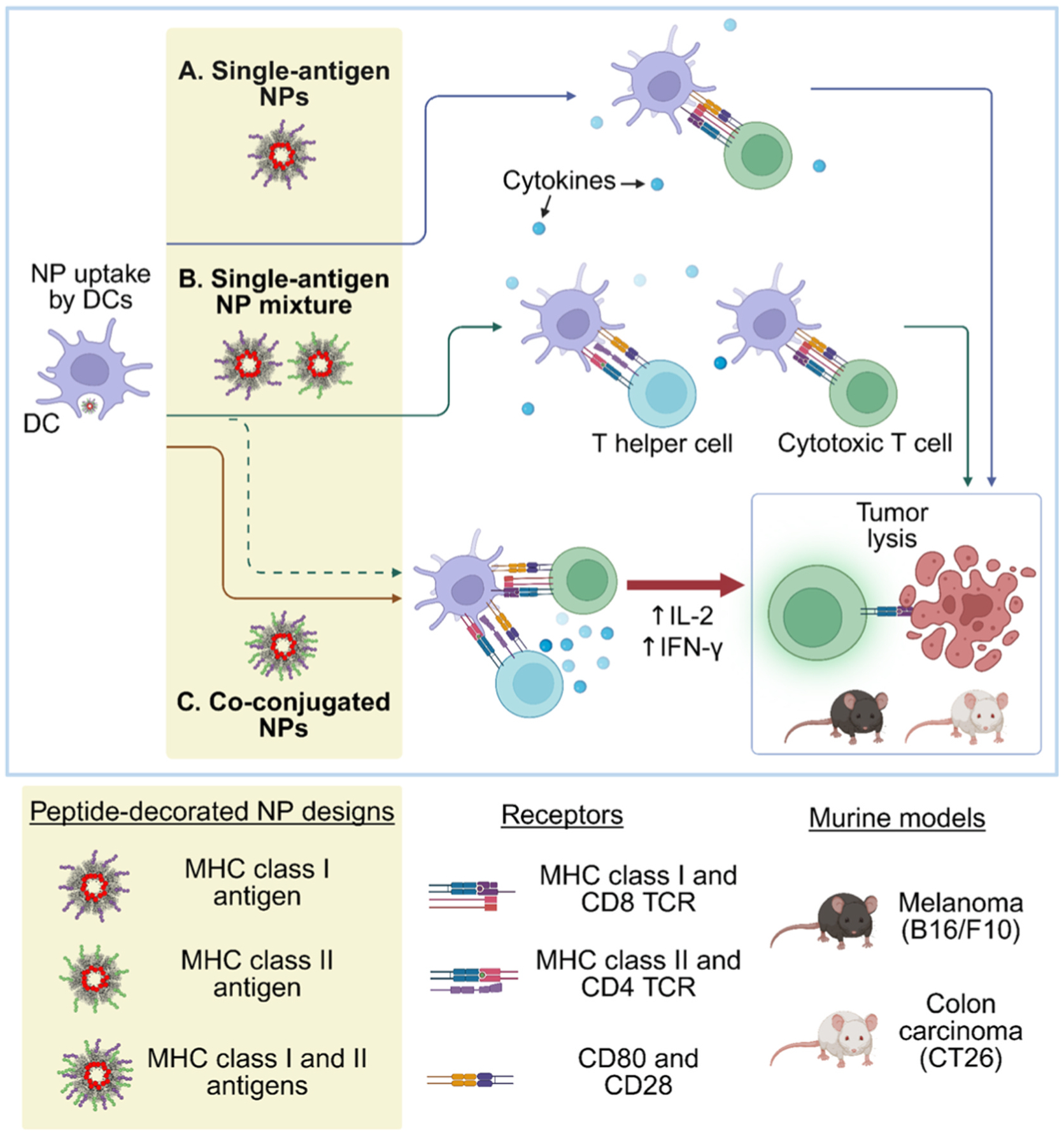
Proposed antigen delivery strategies by which NPs designed to deliver MHC class I and class II antigens can affect anti-tumor immunity through DCs. After NP uptake, DCs may traffic to secondary lymphoid organs (e.g., spleen or LNs), wherein they encounter T cells. **Scenario A:** Single-antigen NPs can be taken up by DCs to activate CD8^+^ T cells. **Scenario B:** Mixture of single-antigen NPs can be taken up by separate DCs and activate CD8^+^ and CD4^+^ T cells. **Scenario C:** Antigens co-conjugated onto NPs can be taken up by individual DCs, which can activate both CD8^+^ and CD4^+^ T cells simultaneously. Facilitated by the cluster of differentiation (CD) CD80:CD28 interaction, DCs which process the co-conjugated NPs may present antigen to both CD8^+^ and CD4^+^ T cells in a spatially and temporally restricted fashion (Scenario C), increasing local concentrations of crucial proliferation, survival, and anti-tumor cytokines such as IL-2 and IFN-γ, and collectively improving cellular immunity and tumor lysis. Within the gray E2 NPs, red represents CpG adjuvant. Purple and green peptides represent MHC class I and MHC class II antigens, respectively. Purple, blue, green, and red cells represent DCs, CD4^+^ T cells, CD8^+^ T cells, and tumor cells, respectively. Small blue dots represent cytokines. NPs were designed, synthesized, and evaluated for the treatment of melanoma (B16/F10 cells in Th1-biased C57BL/6 mice) and colon carcinoma (CT26 cells in Th2-biased BALB/c mice).

DCs are the most potent antigen-presenting immune cells, so-called professional antigen-presenting cells [12]. They activate T cells through presenting antigen in the context of the appropriate MHC molecule and providing co-stimulatory signals [13]. MHC class I molecules display compatible antigenic peptides to cytotoxic T cells (CD8^+^ T cells), while MHC class II molecules present peptides to T helper cells (CD4^+^ T cells) [13]. Cancer vaccine therapies conventionally focus on activating CD8^+^ T cells via presentation of MHC class I antigens to elicit an antigen-specific, cytolytic anti-tumor response [6,14–21]. However, an accumulating body of evidence supports engaging both cytolytic and helper T cell responses [22–24]. The activation of DCs with both sets of T cells elicits several biological responses [12], including facilitating DC maturation through the CD40-CD40L interactions [25], increasing CD8^+^ T cell secretion of pro-inflammatory cytokines (such as interleukin (IL)-2 and interferon (IFN)-γ) [26,27], and promoting cell proliferation (Fig. 1, C) [26–28], relative to the DC-to-CD8^+^ T cell-only interactions (Fig. 1A and B) [25–32]. Despite this evidence supporting the immunological advantage of a DC activating both T cell subsets, the strategy by which NP-based vaccines should deliver MHC class I and II peptides for tumor treatment may influence the magnitude of this collaborative response.

We hypothesized that NP cancer vaccines delivering MHC class I and class II antigens on the same NP would elicit a stronger specific CD8^+^ T cell response and robust anti-tumor response (Fig. 1, C) compared to alternative strategies with MHC class I antigen delivered alone (Fig. 1, A) or separately from MHC class II antigen (Fig. 1, B) on NPs. To test this hypothesis, we synthesized single-antigen NPs (Fig. 1, A), single-antigen NP mixtures (Fig. 1, B), and dual-antigen NPs (Fig. 1, C) for two distinct mouse tumor models [33,34], and we compared the effects of these NP strategies in immunization and tumor treatment studies.

Our previous studies examined a B16/F10 melanoma vaccine using a protein NP (E2) for the simultaneous delivery of Toll-like receptor (TLR) agonist cytosine-phosphorothioate-guanine (CpG) and the MHC class I melanoma peptide gp100_25–33_ (KVPRNQDWL, abbreviated gp100-I); this NP is termed (gp100-I)-CpG-E2 [6,35]. To show efficacy and evaluate robustness of the strategy across different T helper (Th)-polarized backgrounds and MHC haplotypes, the current studies assessed these NP design strategies (Fig. 1) in two syngeneic tumor models, the B16/F10 melanoma and CT26 colon carcinoma models. While B16/F10 is on a Th1-biased C57BL/6 background and has MHC Class I H2^b^ and Class II I-A^b^ haplotype, the CT26 model is on a Th2-biased BALB/c background and has an MHC haplotype of H2^d^ and I-A^d^ [36,37]. We chose the following MHC class I and II antigens for their immunogenicity to be conjugated to our NP platform: gp100-I (gp10025–33, KVPRNQDWL) and gp100-II (gp10045–59, NRQLYPEWTEAQRLD) for the melanoma model, and CT-I (gp70423–431 mimotope, SPSYAYHQF) and CT-II (CT26-ME1 neoantigen, LHSGQNHLKEMAISVLEARACAAAGQS) for the colon carcinoma model [10,38–41].

We used the E2 protein NP vaccine platform for co-delivery of antigen(s) and adjuvant, applying prior conjugation strategies that release both antigen and adjuvant after the NP is taken up by DCs [35,42,43]. E2 NPs consist of 60 identical subunits from pyruvate dehydrogenase and offer a number of advantages, including particle size (~30 nm) within the optimal size range for uptake by DCs, specific functionalization enabled by genetic engineering, and high stability [42,44–46]. Our prior work demonstrated that the co-delivery of vaccine components by attachment to E2 NPs, rather than administering free antigen and adjuvant, improved potency, likely due to enhanced antigen display on activated DCs and reduced off-target effects [35,42,43].

In the present study, we utilized the E2 protein NP platform to evaluate strategies that deliver MHC class I and II antigens to DCs for robust anti-tumor cytotoxic T cell responses, supported by T helper cells (Fig. 1). While C57BL/6 mice are inherently biased towards Th1 responses, BALB/c mice are Th2-biased [47–50]. B16/F10 is an aggressive cancer [48], but it was also important to test anti-CT26 efficacy in the Th2-biased BALB/c mice, where generating Th1 responses is still critical for tumor eradication but could be more difficult to achieve [47,48]. Varying Th biases have been demonstrated in humans, with contributing factors including race, sex, genetics, or psychological state [51–53]. Therefore, developing therapeutic platforms that can elicit robust anti-tumor responses across the spectrum of Th1 and Th2 bias is highly desirable, serving as motivation to evaluate our primary hypothesis (Fig. 1) in multiple murine tumor models.

We synthesized E2 NPs encapsulating CpG adjuvant and decorated with peptides of only MHC class I epitope [(gp100-I)-CpG-E2 or (CT-I)-CpG-E2], only MHC class II epitope [(gp100-II)-CpG-E2 or (CT-II)-CpG-E2], or both classes of epitopes [(gp100-I + II)-CpG-E2 or (CT-I + II)-CpG-E2]. We immunized mice with MHC class I single-antigen NPs, a mixture of MHC class I single-antigen NPs and MHC class II single-antigen NPs, or co-conjugated MHC class I and II antigens on NPs (with equivalent CpG and peptide dosages), and subsequently assessed antigen-specific cytokine secretions (e.g., IFN-γ and IL-2) and splenocyte immune cell population changes. We then evaluated each group for tumor growth and survival in B16/F10 melanoma or CT26 colon carcinoma treatment studies.

## 2. Materials and methods

### 2.1. Materials

All materials were purchased from Fisher Scientific unless otherwise indicated. Peptides were ordered from Genemed Synthesis; these included SIINFEKL, gp100-I (gp100_25–33_, KVPRNQDWL), C-gp100-I (CKVPRNQDWL), gp100-II (gp100_45–59_, NRQLYPEWTEAQRLD), C-gp100-II (CNRQLYPEWTEAQRLD), CT-I (SPSYAYHQF), C-CT-I (CSPSYAYHQF), CT-II (LHSGQNHLKEMAISVLEARACAAAGQS), C-CT-II (CLHSGQNHLKEMAISVLEARACAAAGQS), and C-terminal fluorescently-tagged peptides used to characterize conjugation ratios [FITC-C-gp100-I (K-Fam-tagged CKVPRNQDWL), FITC-C-gp100-II (K-Fam-tagged CNRQLYPEWTEAQRLD), FITC-C-CT-I (K-Fam-tagged CSPSYAYHQF), and TMR-C-CT-II (K-TMR-tagged CLHSGQNHLKEMAISVLEARACAAAGQS)]. Conjugatable aldehyde-modified CpG1826 (tccatgacgttcctgacgtt, abbreviated CpG) was purchased from TriLink.

Culture media used for *in vitro* assays [e.g., IFN-γ enzyme-linked immunospot (ELISpot), IL-2 enzyme-linked immunosorbent assay (ELISA), and Th1/Th2 cytokine analysis via LEGENDplex] consisted of RPMI 1640 (Corning), heat-inactivated fetal bovine serum (10%, Life Technologies), sodium pyruvate (1 mM, HyClone), L-glutamine (2 mM, Lonza), penicillin (100 U/mL, Gibco), streptomycin (100 μg/mL, Gibco), and nonessential amino acids (0.1 mM, Lonza). The B16/F10 melanoma cell culture media contained DMEM (Corning), 10% heat-inactivated fetal bovine serum, 1 mM sodium pyruvate, 2 mM L-glutamine, 100 U/mL penicillin, and 100 μg/mL streptomycin. The CT26 colon carcinoma cell culture media contained RPMI 1640, 10% heat-inactivated fetal bovine serum, 1 mM sodium pyruvate, 2 mM L-glutamine, 100 U/mL penicillin, and 100 μg/mL streptomycin. Antibodies used to stain harvested immune cells from the spleen or lymph nodes (LNs) were purchased from BioLegend and included anti-mouse CD16/32 for blocking non-specific binding, CD11c (FITC) for DCs, F4/80 (APC) for macrophages, B220 (PE) for B cells, and CD3 (PE/Cy7) for T cells, with CD4 (PerCP/Cy5.5 or PerCP) for CD4^+^ T cells and CD8 (AF488 or APC) for CD8^+^ T cells.

### 2.2. Mice

All animal studies were approved by the Institute for Animal Care and Use Committee (IACUC #AUP-22–094) at the University of California, Irvine, which is internationally accredited by the Association for Assessment and Accreditation of Laboratory Animal Care (AAALAC #000238). Female C57BL/6 and BALB/c mice were purchased from Jackson Laboratories at 6–8 weeks of age and housed in standard cages with standard enrichment.

### 2.3. Nanoparticle (NP) syntheses

**Fig. 2.**
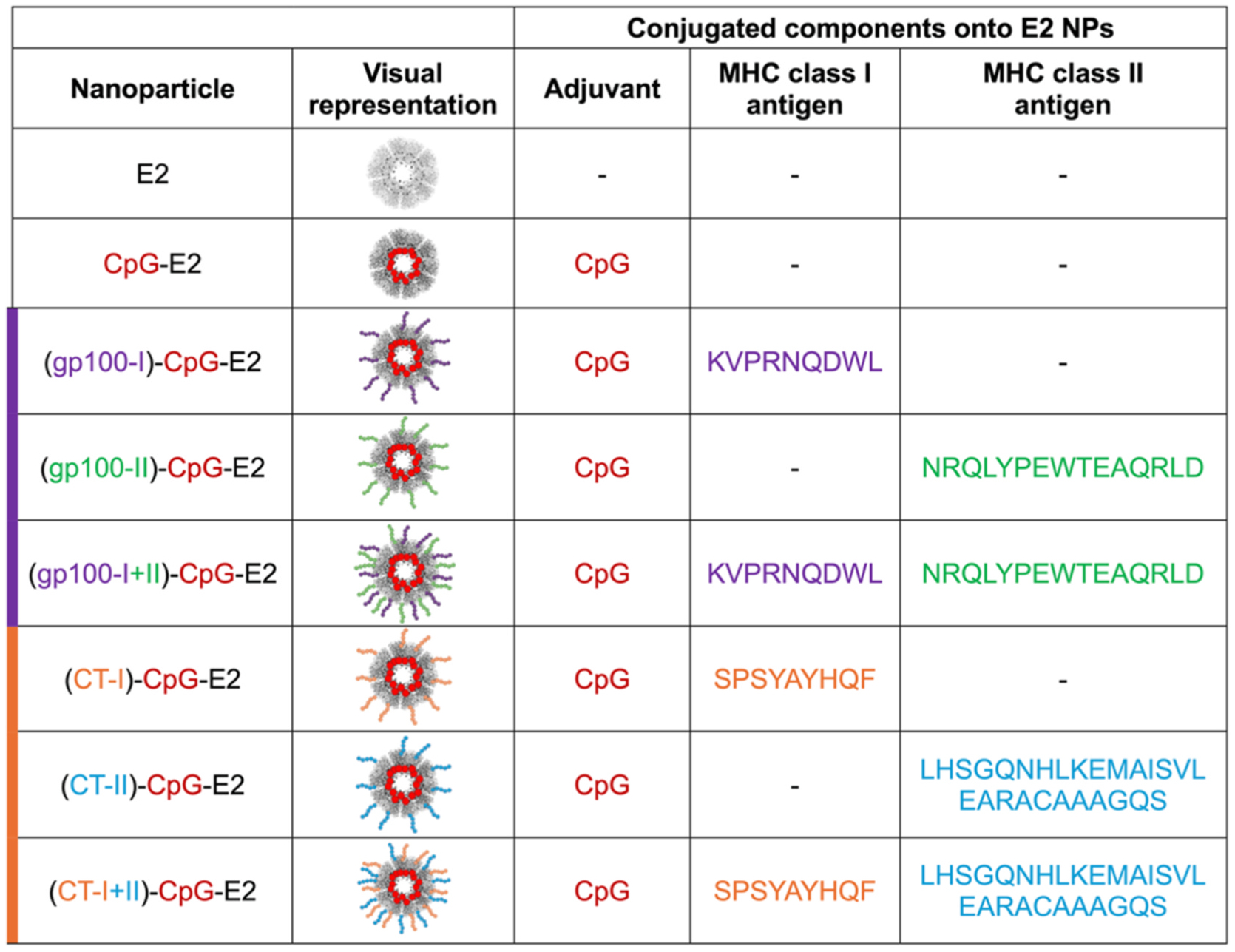
Summary of nanoparticles synthesized to co-deliver CpG and peptide antigens for the melanoma and colon carcinoma models. For each NP design, the visual representation and components are shown. CpG (red) was conjugated to the internal cysteines of E2 NPs (gray). Peptides for the melanoma model [e.g., gp100-I (purple) and gp100-II (green)] [40,55] or peptides for the colon carcinoma model [e.g., CT-I (orange) and CT-II (blue)] [10,41] were conjugated to exposed lysines of E2 NPs. The sequences of gp100-I, gp100-II, CT-I, and CT-II are shown. The ratios of these components per E2 NP monomer following conjugation are described in Table S1. The three rows highlighted by the purple and orange bars are relevant for the melanoma and colon carcinoma models, respectively.

The NPs used in this study are summarized in Fig. 2. To obtain the NP scaffolds, E2 (D381C E2) protein NPs were purified from *E. coli* lysates using previously published protocols [35,45,54]. Characterization of NPs included a combination of dynamic light scattering (DLS), sodium dodecyl sulfate-polyacrylamide gel electrophoresis (SDS-PAGE) analysis (Fig. S1), bicinchoninic acid assay (BCA), and transmission electron microscopy (TEM). To synthesize CpG-E2 NPs, the conjugation of CpG to the internal hollow E2 NP cavity was performed with a cysteine-reactive BMPH (N-(β-maleimidopropionic acid)) linker, as previously described [42,44]. Non-reacted adjuvant was removed over 24 h with dialysis into phosphate buffer (50 mM potassium phosphate, 100 mM NaCl, pH 7.4) using 300 kDa Float-A-Lyzer G2 Dialysis Devices (Spectrum Laboratories).

To decorate the exposed lysines of E2 NPs with only one MHC class of antigen, conjugation protocols were adapted from previously described methods [6,35,42–44,56]. E2 was incubated with 20-fold molar excess of 4-(N-maleimidomethyl)-cyclohexane-1-carboxylate (sulfo-SMCC) to E2 monomer for 30 min at room temperature, followed by removal of excess linker using 7 kDa cutoff Zeba desalting columns (Pierce). Cysteine-modified peptides were reduced with 10-fold molar excess of tris(2-carboxyethyl)phosphine (TCEP) for 30 min at room temperature prior to incubation with NPs. To synthesize (gp100-I)-CpG-E2, (gp100-II)-CpG-E2, (CT-I)-CpG-E2, or the fluorescently-tagged NPs FITC-(gp100-I)-E2, FITC-(gp100-II)-E2, or FITC-(CT-I)-E2, a 10-fold molar excess of reduced peptides to E2 monomer was reacted for 2 h at room temperature, followed by removal of unreacted peptides over 24 h by dialysis in phosphate buffer using 300 kDa Float-A-Lyzer G2 Dialysis Devices (Spectrum Laboratories). Similar conjugation protocols were used for (CT-II)-CpG-E2 and TMR-(CT-II)-E2 conjugations, except that a 2-fold molar excess of reduced peptides to E2 monomer was used. To determine the peptide-to-NP ratio, the peptide concentration was determined by measuring absorbance at 500 nm and 555 nm for the resulting FITC-labeled NPs and TMR-labeled NPs, respectively, while the E2 concentration was obtained by BCA assay.

For dual-antigen NPs, protocols were modified to co-conjugate both MHC class I and II peptides onto E2 NPs with sulfo-SMCC linker. Cysteine-modified peptide mixtures were first reduced with 10-fold molar excess of TCEP for 30 min at room temperature. To synthesize (gp100-I + II)-CpG-E2 NPs or (CT-I + II)-CpG-E2 NPs, a 1:2:1 molar ratio of C-gp100-I: C-gp100-II: E2 monomer or 1:3.5:1 molar ratio of C-CT-I: C-CT-II: E2 monomer, respectively, was utilized. To determine resulting conjugation ratios, only one MHC class of peptide per mixture was fluorescently-tagged to perform absorbance readings at 500 nm or 550 nm for one species at a time. For instance, to characterize the amount of gp100-I conjugated in a co-conjugated NP, E2 was reacted with FITC-C-gp100-I and C-gp100-II. The average number of peptides conjugated per NP was calculated by dividing the molar peptide concentration by the molar E2 concentration. TEM was performed by staining dual-antigen NPs with 2% uranyl acetate on carbon-coated copper grids (Ted Pella) and imaging on a JEM-2100F electron microscope.

Lipopolysaccharides (LPS) were removed using Triton X-114, and limulus amebocyte lysate test (LAL) was used to confirm that there were <5 EU per milligram of E2 prior to CpG and peptide conjugation, consistent with previous studies [35,42–44]. All NPs, including purified E2, CpG-E2, and antigen-conjugated CpG-E2 NPs were stored in phosphate buffer.

### 2.4. Immunizations

Mice were immunized subcutaneously, bilaterally at both flanks on days 0 and 7 with E2 NPs containing CpG and displaying melanoma antigens (gp100-II and/or gp100-I peptides for C57BL/6 mice), E2 NPs containing CpG and displaying colon carcinoma antigens (CT-II and/or CT-I peptides for BALB/c mice), or PBS as a control. A summary of all groups and dosages for each component, including E2, CpG, and antigen, is described in Figs. 5B and 8B, and S2A. To enable comparisons between groups, some formulations were adjusted with addition of CpG-E2 to obtain equivalent amounts of E2 and CpG, as needed. Experiments were terminated on day 14, and spleens and LNs were harvested (Fig. S2B–D). For studies using C57BL/6 mice immunized with NPs decorated with melanoma antigens, spleens were homogenized, depleted of red blood cells using ACK lysing buffer (Gibco). Cells isolated from these lymphoid tissues routinely exhibited 90%-95% viability, as assessed by trypan blue (Gibco) exclusion. Following isolation, samples were analyzed with a combination of carboxyfluorescein succinimidyl ester (CFSE, eBioScience) staining, flow cytometry, ELISA, or ELISpot assay. Immunization studies in the BALB/c model using NPs displaying the colon carcinoma peptides similarly included a combination of IFN-γ ELISpot, flow cytometry, and cytokine analysis. Flow cytometry required blocking with anti-mouse CD16/32 for 10 min to reduce non-specific staining, followed by anti-mouse antibody staining for (set 1) CD11c, F4/80, and B220 or (set 2) CD3, CD4, and CD8 for 30 min on ice. The gating strategy is described in Fig. S3A–C.

### 2.5. CFSE proliferation

To quantify the proliferation of overall T cells, CD8^+^ T cells (CD3^+^CD8^+^), and CD4^+^ T cells (CD3^+^CD4^+^) in response to peptide recall, splenocytes were stained with 5 μM CFSE in PBS for 10 min at room temperature, washed 3 times with a 5-fold excess volume of ice-cold media, centrifuged at 300×*g* for 5 min, and re-suspended in 500 μL of media.

CFSE-stained splenocytes (or non-stained controls) were plated into 96-well tissue culture-treated, U-bottom plates at 200,000 cells/well. Cells were incubated with a specific condition, such as PBS (negative control), 5 μg/mL ConA (concanavalin A, a potent mitogen [57] as positive control), or 50 μg/mL of peptide, for 72 h at 37 °C. Relevant peptides included gp100-I or gp100-II (without cysteine modification), while irrelevant peptide SIINFEKL was used as a control. Afterwards, cells were collected for flow cytometry. Non-specific antibody binding was reduced with anti-mouse CD16/32 antibody for 10 min on ice, followed by washing with fluorescence-activated cell sorting buffer (PBS with 1% bovine serum albumin (BSA) and 0.1% sodium azide), centrifugation at 300×*g* for 5 min, and discarding of supernatant. For immunization studies using dual-antigen NPs, cells were then stained for CD3, CD4, and CD8 expression using PE/Cy7, PerCP, or APC fluorescent antibodies, respectively. The FITC channel was used for the CFSE stain.

Non-proliferated cells or daughter generations were assessed within all cells, CD4^+^ T cell, or CD8^+^ T cell populations, as described in Fig. S3. Relative proliferation ratio G_n_/G_0_ following a specific incubation condition was calculated as the number of proliferated cells (generations n > 0; G_n_) divided by the number of non-proliferated cells (generation 0; G_0_), and normalized to the analogous ratio for non-stimulated control for the same biological replicate:

$$\frac{{\text{G}}_{\text{n}}}{{\text{G}}_{0}}=\frac{{\left(\frac{{\text{N}}_{0}}{\sum_{1}^{\text{n}}{\text{N}}_{\text{i}}}\right)}_{\text{incubation}}}{{\left(\frac{{\text{N}}_{0}}{\sum_{1}^{\text{n}}{\text{N}}_{\text{i}}}\right)}_{\text{PBS}}}$$

where G_n_/G_0_ is the relative proliferation ratio, N_0_ is the initial number of cells, N_i_ is the number of cells in generation (G) number i, and n is the total number of generations. Incubation conditions included SIINFEKL, ConA, and relevant peptides.

### 2.6. IFN-γ ELISpot

IFN-γ ELISpot assays were performed as we have previously described [56]. High binding 96-well plates (Millipore) were coated overnight with IFN-γ capture antibody (Pharmingen). After ACK lysing buffer treatment and quenching as described above, 800,000 or 500, 000 cells from each spleen of the C57BL/6 or BALB/c mice, respectively, were plated per well. Cells were incubated overnight (~18 h) with 1 μg of peptide (SIINFEKL, gp100-I, gp100-II, CT-I, or CT-II) or 0.5 μg ConA as a positive control. After incubation, conditioned media was recovered for IL-2 ELISA analysis.

IFN-γ ELISpot plates were washed with PBS-T (PBS with 0.05% Tween 20) or PBS, incubated with a biotinylated detection antibody (Pharmingen) in 1% BSA in PBS-T for 1 h, incubated with streptavidin-alkaline phosphatase diluted in 1% BSA in PBS for 30 min, incubated with 1-step NBT/BCIP Substrate Solution (Thermo Scientific), and stopped by washing extensively with water. After air-drying overnight, plates were scanned and analyzed using an ELISpot plate reader (Cellular Technology) and an immunospot analysis software package (Immunospot Analysis Pack).

### 2.7. IL-2 ELISA

To determine IL-2 secretion levels, an IL-2 ELISA kit (BioLegend) was used according to the recommended protocols. For IL-2 standard (BioLegend), 2-fold dilutions of standard were prepared. For processing, IL-2 ELISA plates were washed with wash buffer (PBS-T), coated with capture antibody in coating buffer (8.4 g NaHCO_3_, 3.56 g Na_2_CO_3_, in 1.0 L of water at pH 9.5), blocked for non-specific binding with assay diluent (PBS with 1% BSA) for 1 h, incubated with samples or standard for 2 h, incubated with detection antibody for 1 h, incubated with avidin-horseradish peroxidase solution for 30 min, incubated with TMB substrate solution (BioLegend) in the dark, and stopped with 2 N H_2_SO_4_. Absorbance at 450 nm was immediately read by plate reader. Per-plate standard curves were generated with seven titrations of IL-2 standard. A 5-parameter logistics curve-fitting algorithm was used to correlate IL-2 concentration to absorbance for each standard curve.

### 2.8. Th1 and Th2 cytokine analyses

To analyze the concentrations of cytokines (IFN-γ, tumor necrosis factor (TNF)-α, IL-2, IL-4, IL-5, IL-6, IL-10, and IL-13) secreted by splenocytes stimulated with CT-I, CT-II, and SIINFEKL, we utilized the LEGENDplex MU Th1/Th2 Panel (8-plex) (BioLegend). Per well in a 96-well plate, 500,000 splenocytes were plated and stimulated with 1 μg of peptide (CT-I, CT-II, or SIINFEKL) overnight. The supernatant was then collected for cytokine analysis following the manufacturer's recommended protocol. The final cytokine concentrations were determined using the LEGENDplex Data Analysis Software Suite after data acquisition on a flow cytometer (NovoCyte 3000).

### 2.9. B16/F10 melanoma tumor treatment

For the melanoma model, 6–8-week-old female C57BL/6 mice were subcutaneously inoculated with 1 × 10^4^ B16/F10 tumor cells (ATCC, Cat. CRL-6475) in the right flank, defined as day 0 of tumor treatment studies. On days 1 and 8, mice were subcutaneously, bi-laterally treated with NPs or PBS negative control (Fig. 6A). The treatment groups are summarized in Fig. 6B and include: PBS (Group 1); (gp100-I)-CpG-E2 with CpG-E2 (Group 2); (gp100-I)-CpG-E2, (gp100-II)-CpG-E2, and CpG-E2 (Group 3); and (gp100-I + II)-CpG-E2 (Group 4). The amounts of all components for each group are described in Fig. 5B and S2A. To enable comparisons between groups, Groups 2 and 3 were adjusted with the addition of CpG-E2 to obtain equivalent amounts of E2 and CpG. Tumor size was measured every day with a caliper, and the volume was approximated as [0.5 × (shortest diameter)^2^ × longest diameter]. Palpable and terminal tumor volumes were ~50 mm^3^ and 1000 mm^3^, respectively.

### 2.10. CT26 colon carcinoma treatment

For the CT26 colon carcinoma model, 6–8-week-old female BALB/c mice were subcutaneously inoculated with 5 × 10^4^ CT26 tumor cells (ATCC, Cat. CRL-2638) in the right flank, defined as day 0 of the tumor treatment studies. On days 3 and 10, mice were subcutaneously, bilaterally treated with NPs or PBS negative control (Fig. 9A). The experimental groups are summarized in Fig. 9B and include: PBS (Group 1); (CT-I)-CpG-E2 with CpG-E2 (Group 2); (CT-I)-CpG-E2 and (CT-II)-CpG-E2 (Group 3); and (CT-I + II)-CpG-E2 with CpG-E2 (Group 4). The amounts of each component are also described in Fig. 8B and S2A. To enable comparisons between groups, Groups 2 and 4 were adjusted with the addition of CpG-E2 to obtain equivalent amounts of E2 and CpG. Tumor volumes were measured daily with a caliper, as described above.

### 2.11. Statistical analyses

At least three independent syntheses were performed to characterize NP sizes and component conjugation ratios (e.g., components CpG, gp100-I, and gp100-II; or CpG, CT-I, and CT-II), with measurements presented as the mean ± standard deviation (SD). Immunization studies utilized N ≥ 3 biological replicates per group. For CFSE and IFN-γ assays, each biological replicate had 3 technical replicates per incubation condition, while for LEGENDplex analysis, each biological replicate had 2 technical replicates per condition. Data were presented as mean ± standard error of the mean (SEM) unless noted otherwise. Flow cytometry to determine immune populations in the spleen or LNs was analyzed using one-way ANOVA with post-hoc Bonferroni's test. CFSE, IFN-γ ELISpot, and Th1/Th2 cytokine assays were analyzed using twoway ANOVA with post-hoc Bonferroni's test unless noted otherwise. The B16/F10 tumor treatment study utilized N = 10 biological replicates per group, while the CT26 tumor treatment study used N ≥ 7 biological replicates per group, with tumor survival assessed with log-rank tests. P-values less than 0.05 were considered statistically significant.

## 3. Results

### 3.1. E2 protein NPs displaying MHC class I and II epitopes for the melanoma and colon carcinoma models were synthesized and characterized

In these studies, several different NPs were synthesized by conjugating the E2 NP with the CpG adjuvant and MHC class I and II antigens from the B16/F10 melanoma or CT26 colon carcinoma models. As summarized in Fig. 2, CpG-E2 NPs encapsulated CpG but were not conjugated to antigen peptides. Antigens were attached to these CpG-E2 NPs to examine the response of CD8^+^ and CD4^+^ T cells following immunization; melanoma antigens included MHC class I and class II peptides derived from gp100 TAA (abbreviated gp100-I and gp100-II peptides, respectively), conjugated to NPs individually [yielding (gp100-I)-CpG-E2 and (gp100-II)-CpG-E2], or co-conjugated together on NPs [yielding (gp100-I + II)-CpG-E2]. Similarly, colon carcinoma antigen peptides included MHC class I and class II peptides (abbreviated CT-I and CT-II, respectively), and single-antigen NPs [yielding (CT-I)-CpG-E2 and (CT-II)-CpG-E2)] or dual-antigen NPs [yielding (CT-I + II)-CpG-E2] were synthesized. The linkers used for attaching CpG and peptides to E2 NPs were acid-hydrolyzable and/or degradable *in vivo*, respectively, allowing the components’ release in endosomal environments of DCs [42].

**Fig. 3.**
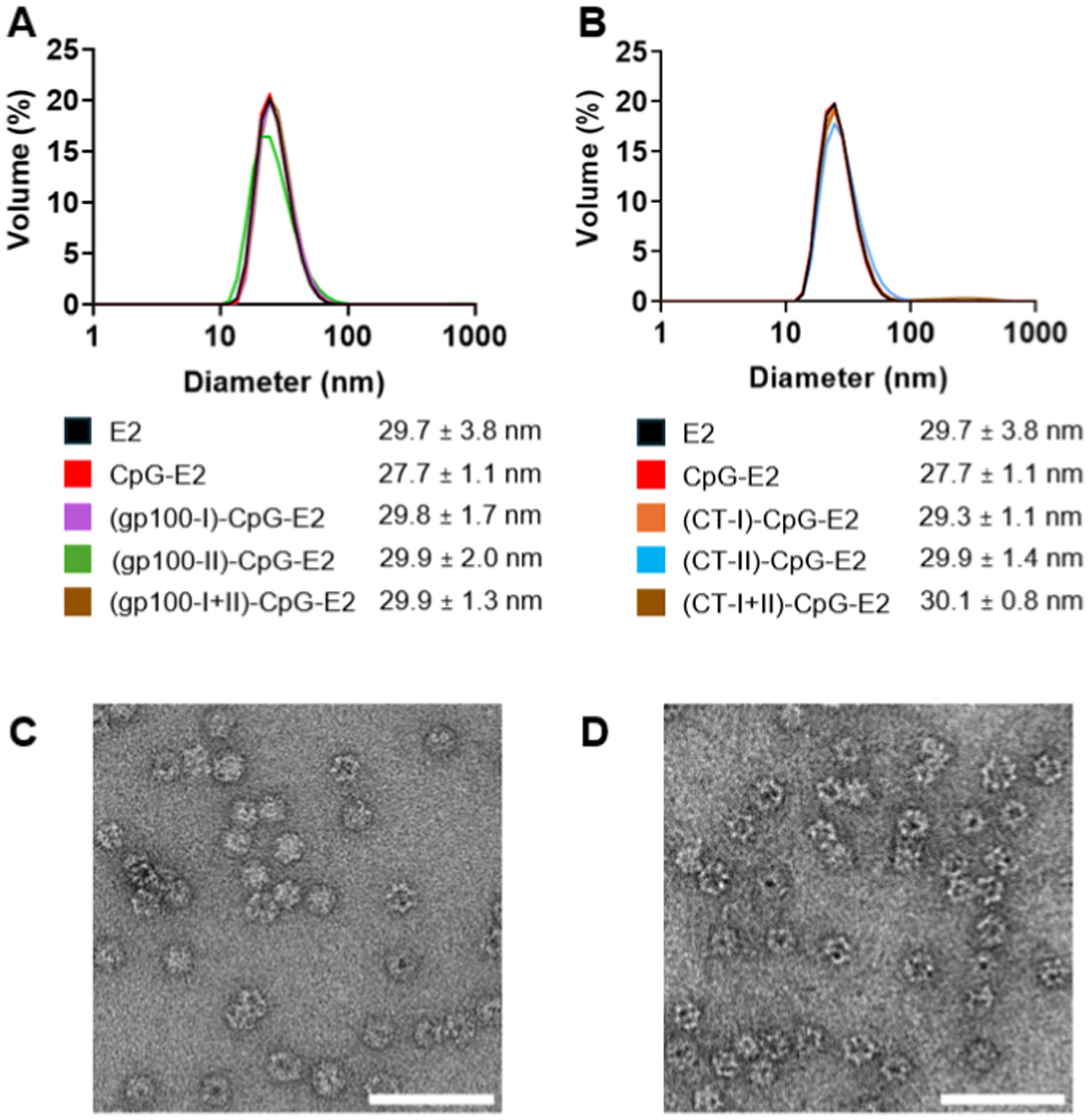
E2 NPs decorated with gp100 or CT peptides maintained NP structure and solubility. **(A)** Representative DLS and average hydrodynamic diameters of E2 NPs relevant to gp100 melanoma antigens [E2, CpG-E2, (gp100-I)-CpG-E2, (gp100-II)-CpG-E2, and (gp100-I + II)-CpG-E2]. *Mean* ± *SD. N*=*3*. **(B)** Representative DLS and average hydrodynamic diameters of E2 NPs relevant to CT26 colon carcinoma antigens [E2, CpG-E2, (CT-I)-CpG-E2, (CT-II)-CpG-E2, and (CT-I + II)-CpG-E2]. *Mean* ± *SD. N*=*3*. **(C**–**D)** Representative TEM image of **(C)** (gp100-I + II)-CpG-E2 NPs and **(D)** (CT-I + II)-CpG-E2 NPs. Scale bars = 100 nm.

All nanoparticles, including E2, CpG-E2, (gp100-I)-CpG-E2, (gp100-II)-CpG-E2, (gp100-I + II)-CpG-E2, (CT-I)-CpG-E2, (CT-II)-CpG-E2, and (CT-I + II)-CpG-E2, exhibited hydrodynamic diameters of ~30 nm, as measured by DLS (Fig. 3A and B). These NP sizes are within the optimal size range for endocytosis by DCs [58,59], the most potent antigen-presenting cells. We previously demonstrated that E2 protein NPs containing CpG, with diameters ~30 nm, are taken up by and activate both major classes of DCs (myeloid and plasmacytoid DCs) and enable increased amounts of antigen display [44]. The intact and symmetric structure of NPs was confirmed by TEM, following the conjugation of CpG, gp100-I, and gp100-II to yield (gp100-I + II)-CpG-E2 NPs (Fig. 3C), and CpG, CT-I, and CT-II to yield (CT-I + II)-CpG-E2 NPs (Fig. 3D). The structural stability of these NPs was expected, as we have previously demonstrated that the E2 NP scaffold remains stable and intact up to 80 °C [45]. Furthermore, this stability is retained following ligand conjugation on the surface and molecular attachment in the internal cavity for drug delivery, cell targeting, and immunological studies [44–46,54,60–62].

The conjugation of CpG and antigen peptides to E2 monomers was confirmed and quantified (Fig. S1; Table S1). E2 NPs are self-assembled from 60 identical monomers, and the theoretical molecular weight of a single E2 subunit monomer is 28.1 kDa, observable as a band on SDS-PAGE gels (Fig. S1, lane 2). The conjugation of a CpG molecule via the linker to the single cysteine of an E2 monomer would increase the theoretical molecular weight of the monomer to 34.9 kDa, which correlates to the observable shift in a fraction of the monomers following CpG conjugation reactions (Fig. S1, lane 3). Based on these intensities, an average of 19 ± 2 CpG molecules were conjugated per 60-mer E2 NP, consistent with our previously published studies [44,60].

Broad bands were observed on SDS-PAGE for E2 NPs after single-(MHC class I peptide or MHC class II peptide) or dual-antigen (MHC class I + II peptides) conjugation for both the melanoma and colon carcinoma models, indicative of a range of peptide numbers conjugated per E2 monomer (Fig. S1, lanes 4–6), consistent with previous E2 NP formulations [6,35,42–44]. To quantify the conjugation ratio, we used fluorescently-tagged peptides; for single-antigen NPs, the number of peptides displayed was on average ~214 for gp100-I, ~129 for gp100-II, ~209 for CT-I, and ~83 for CT-II, per 60-mer E2 NP (Table S1).

Target antigen ratios were based on *in vivo* doses that could elicit a biological effect, described below in sections 3.2.1–3.2.2 and 3.3.1–3.3.2. For the dual-antigen NPs, we aimed for a molar ratio of melanoma peptides gp100-I: gp100-II of 1:2, yielding an actual number of 42 ± 11 gp100-I and 73 ± 20 gp100-II peptides conjugated to a 60-mer E2 NP [abbreviated (gp100-I + II)-CpG-E2] (Table S1). Similarly, for colon carcinoma peptides, the target molar ratio of CT-I: CT-II on dual-antigen NPs was 1:3, yielding 39 ± 2.6 CT-I and 111 ± 9.4 CT-II peptides per 60-mer E2 NP [abbreviated (CT-1+II)-CpG-E2] (Table S1).

### 3.2. Nanoparticle vaccine investigations for the B16/F10 melanoma model

Our previous work examined vaccines comprised of MHC class I antigens on NPs [(gp100-I)-CpG-E2] for the prevention and treatment of B16/F10 melanoma in C57BL/6 mice [6,35]. Immunization with (gp100-I)-CpG-E2 NPs induced antigen-specific activation and proliferation of CD8^+^ T cells and increased survival in tumor-bearing mice [6, 35]. However, we had not investigated the delivery of MHC class II melanoma antigens (e.g., gp100-II) by NPs, either alone or in combination with the MHC class I peptide (gp100-I). We expected that the co-delivery of these antigens by NP vaccines would be important for facilitating CD4^+^ T cell support of CD8^+^ T cell activity (Fig. 1, C), and these results are described below.

#### 3.2.1. Immunization with single-antigen gp100-II NPs induced antigen-specific proliferation

**Fig. 4.**
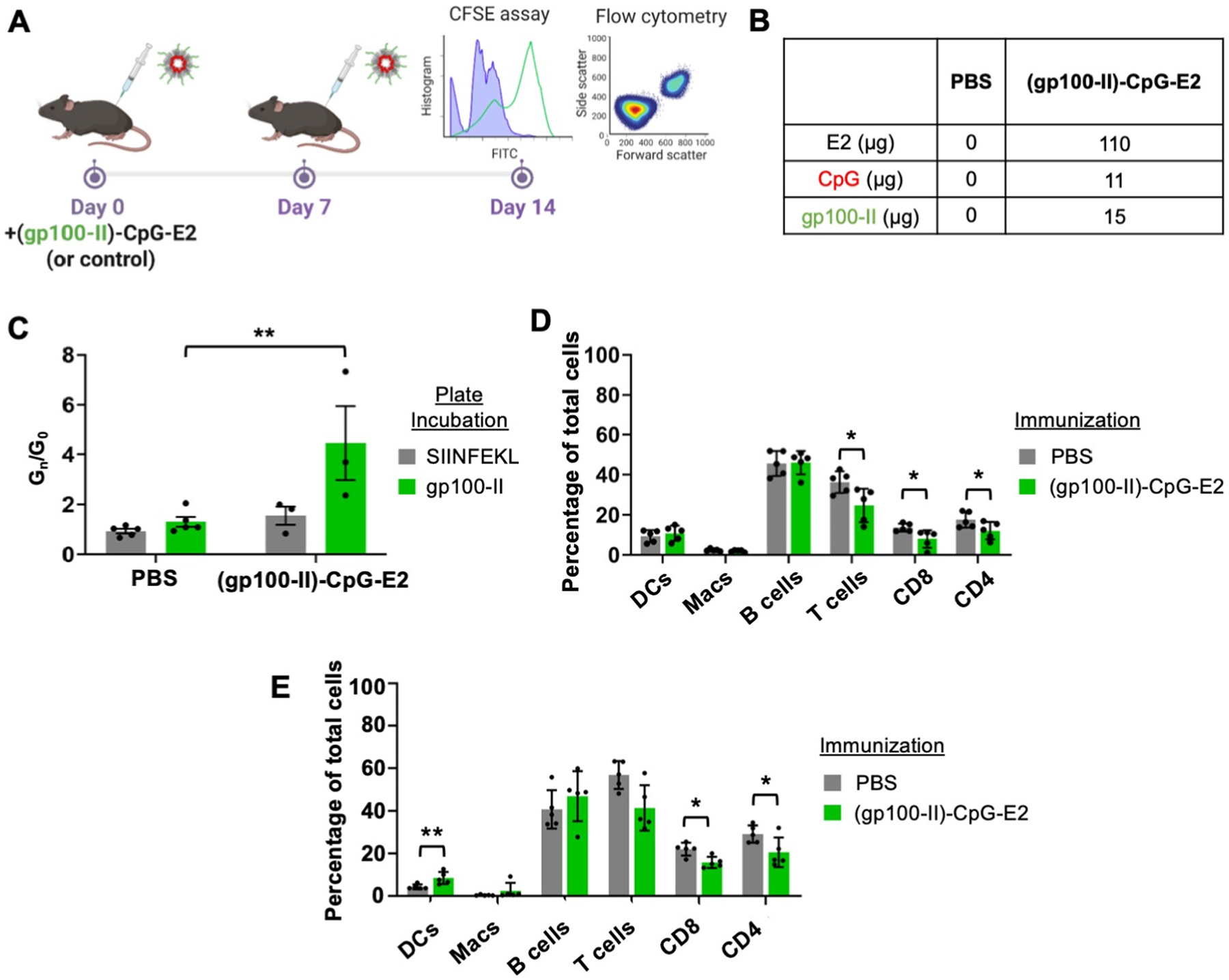
Immunization with E2 NPs displaying MHC class II melanoma TAA induced antigen-specific splenocyte proliferation and modulated immune populations. **(A)** Immunization and experimental schedule. Flow cytometry analysis was performed on day 14. For the CFSE assay, cells were labeled on day 14 and analyzed 72 h later (day 17). **(B)** Nanoparticle vaccine components per immunization dose, including the amount of E2, CpG, and gp100-II peptide per dose. **(C)** Normalized ratio of proliferated versus non-proliferated CFSE-stained splenocytes incubated with gp100-II or irrelevant peptide SIINFEKL (control). *Mean* ± *SEM. N*≥*3. Statistics: Two-way ANOVA with post-hoc Bonferroni's test*. **(D**–**E)** Percentages of DCs, macrophages, B cells, T cells, CD8^+^ T cells, and CD4^+^ T cells, from **(D)** splenocytes and **(E)** lymph nodes after immunization with (gp100-II)-CpG-E2 NPs or PBS. *Mean* ± *SD. N*=5. Statistics: One-way ANOVA with post-hoc Bonferroni's test. *p ≤ 0.05, **p ≤ *0.01*.

We first examined whether immunization with the MHC class II antigen (gp100-II) on NPs, without MHC class I antigen, could specifically educate CD4^+^ T cells. C57BL/6 mice were immunized with (gp100-II)-CpG-E2 NPs (Fig. 4A and B), and we quantified antigen-specific splenocyte proliferation (Fig. 4C and S3D) and immune cell populations in the spleen and lymph nodes (LNs) (Fig. 4D and E). Given our previous demonstrations of dendritic cell activation and maturation, as well as robust MHC class I antigen-specific T-cell responses using the E2 NP platform, we chose to focus on examining MHC class II antigen-specific T cell functional responses [6,35,42]. Studies have found that following TCR engagement with DCs, CD4^+^ T cells produce lower levels of IFN-γ than CD8^+^ T cells [63]; therefore for these immunization studies with (gp100-II)-CpG-E2 NPs (without MHC class I antigen peptides), we performed CFSE proliferation assay to determine the functional activation of T cells rather than measuring IFN-γ levels.

Our prior studies consistently showed that E2 NP immunotherapies elicited the highest immune responses (e.g., specific IFN-γ secretion, T cell-mediated tumor cell lysis, and DC activation) when both antigen and adjuvant were attached onto the NP (antigen-CpG-E2), compared to conditions in which any of the individual components remained unbound to the NP (e.g., antigen-E2 [NP] + CpG; antigen + CpG-E2 [NP]; antigen + CpG + E2 [NP]; etc.) [35,42,43,64]. Furthermore, these immune responses elicited by the E2-based immunotherapies were always antigen-specific [6,35,42,43]. Due to the consistent results of these controls, irrespective of the tumor-associated antigens investigated, similar unbound controls were not performed here to minimize animal numbers.

Splenocytes from animals immunized with (gp100-II)-CpG-E2 NPs proliferated when incubated with gp100-II but not with irrelevant peptide SIINFEKL, demonstrating a 3.4-fold increase in the normalized ratio of proliferated versus non-proliferated (G_n_/G_0_) cell numbers (Fig. 4C). This was consistent with the slightly larger spleens and LNs from the NP group compared with the PBS group (Fig. S2B and S2C, Groups B vs. A). Phenotypical analysis of immune cell populations showed significant reductions in the percentages of CD8^+^ and CD4^+^ T cells in the spleen (Fig. 4D) and LNs (Fig. 4E) from the mice immunized with (gp100-II)-CpG-E2 NPs. These mice also had higher DC percentages in the LNs compared to PBS (Fig. 4E). We note that previous melanoma immunotherapy studies in murine models have used 100–200 μg of MHC class II antigenic peptide per dose and 5–20 μg of CpG adjuvant per dose (both free in solution; no NPs); this is an order of magnitude higher than the antigen levels used here in our investigation to yield an immune response, demonstrating the increased efficacy due to NP attachment of the antigen and adjuvant [65,66].

#### 3.2.2. Co-delivery of MHC class I and II gp100 epitopes on dual-antigen NPs induced robust antigen-specific Th1 responses and T cell proliferation

**Fig. 5.**
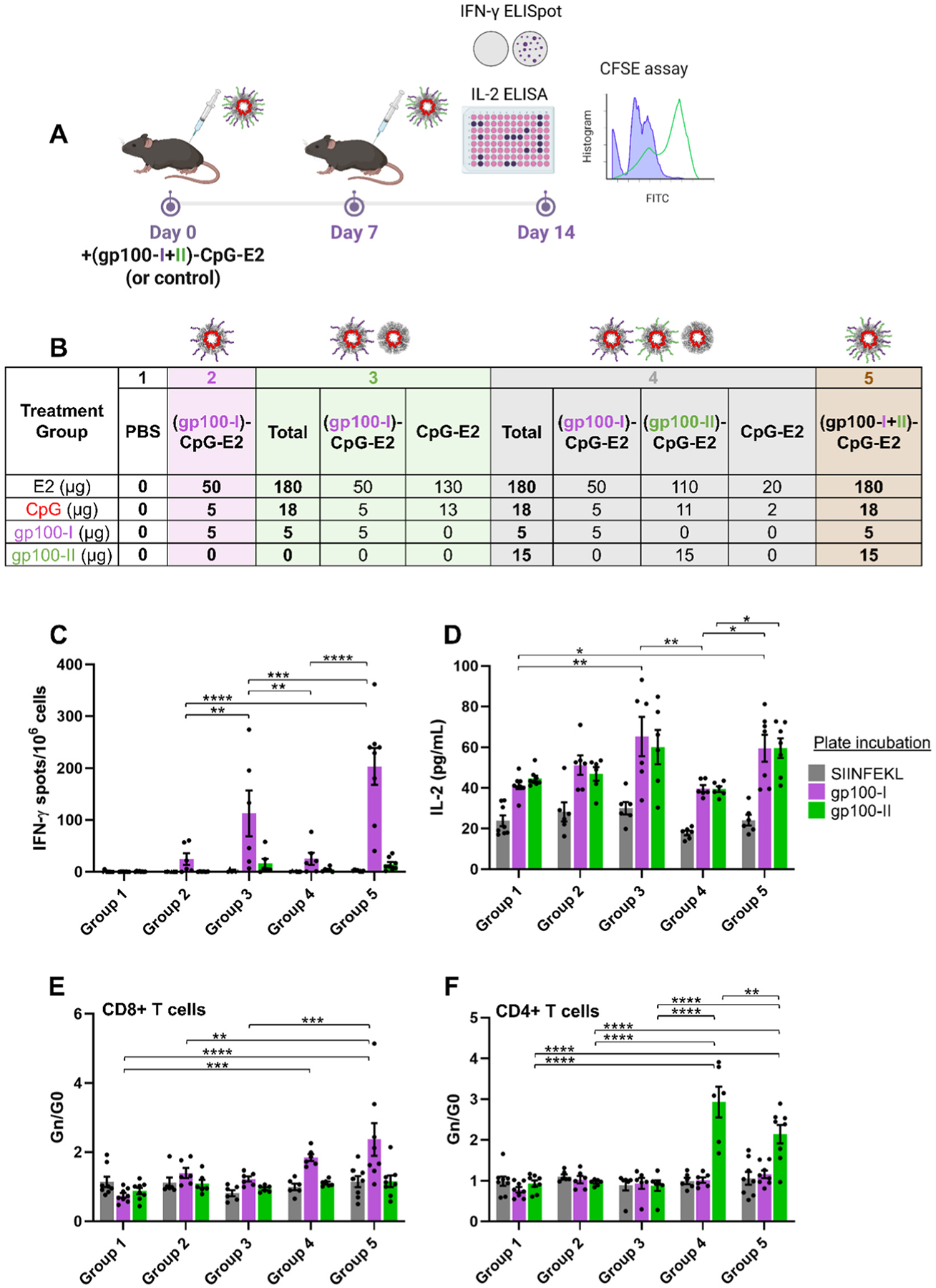
Immunization with E2 NPs displaying MHC class I or class II melanoma TAAs induced antigen-specific Th1 responses and T cell proliferation. **(A)** Immunization schedule. **(B)** Nanoparticle vaccine components (E2 NP, CpG adjuvant, and peptides) per immunization dose. CpG-E2 was included in Groups 3 and 4 to match E2 and CpG doses of Group 5. **(C)** IFN-γ producing spots per million splenocytes, incubated with peptide indicated (SIINFEKL, gp100-I, or gp100-II). **(D)** IL-2 concentrations, in conditioned media from splenocytes incubated with peptide. **(E, F)** Normalized ratios of proliferated versus non-proliferated splenic **(E)** CD3^+^CD8^+^ cells (CD8^+^ T cells), or **(F)** CD3^+^CD4^+^ cells (CD4^+^ T cells), following 72 h incubation with the peptide indicated. Gray, purple, and green bars represent incubation with SIINFEKL (irrelevant peptide), gp100-I, and gp100-II, respectively. *Mean* ± *SEM. N*≥6, 3 technical replicates. Statistics: Two-way ANOVA with post-hoc Bonferroni's test. *p ≤ 0.05, **p ≤ 0.01, ***p ≤ 0.001, ****p ≤ *0.0001*.

We examined our hypothesis that immunization with NPs co-delivering MHC class I and II antigens [(gp100-I + II)-CpG-E2; Group 5] would elicit the greatest increase in specific immunity, relative to conventional MHC class I peptide-bound NPs alone [Groups 2 and 3] or mixtures of MHC class I peptide-bound NPs with MHC class II peptide-bound NPs [Group 4] (Fig. 5). To enable comparisons, antigen and adjuvant doses were equivalently matched between groups where possible (Fig. 5B), and the same amount of gp100-I in all NP groups [Groups 2–5] were used as in our previous studies [6,35]. The amount of gp100-II peptide per dose [Groups 4–5] (Fig. 5A and B) was also the same as for studies in Fig. 4. Within these groups, the CpG dose used in Group 2 was selected to match our prior studies and served as a comparative control [6,35]. Group 3 was included to match the amounts of MHC class I antigen, CpG, and E2 in the co-conjugated NP group [Group 5], which required a higher CpG dosage due to conjugation constraints of the co-conjugated NP.

In splenocytes harvested from mice that were immunized with the dual-antigen NP [(gp100-I + II)-CpG-E2; Group 5], both IFN-γ (Fig. 5C) and IL-2 (Fig. 5D) were secreted at the highest levels relative to other groups. After gp100-I incubation, splenocytes collected from Group 5 yielded an average 1.8- and 8.0-fold higher IFN-γ secretion spot formation compared to those from Group 3 (gp100-I only) and Group 4 (with gp100-I and gp100-II, but delivered by separate NPs), respectively (Fig. 5C). Group 3 gave 4.5-fold more IFN-γ-producing splenocyte spots on average than Group 2, likely due to a higher amount of CpG, as the amount of MHC class I peptide was kept consistent, and we have previously shown that the E2 NP does not contribute to T cell proliferation or IFN-γ responses [35,43]. No group yielded gp100-II-specific IFN-γ levels that were above background. Average secreted IL-2 levels were highest for immunizations with dual-antigen NPs [Group 5] and with single-antigen gp100-I NPs [Group 3] (Fig. 5D).

The greatest and second-greatest increase in CD8^+^ and CD4^+^ T cell proliferation, respectively, was observed after immunization with dual-antigen NPs containing both gp100-I and gp100-II peptides [Group 5] (Fig. 5E and F). The normalized proliferation ratio (G_n_/G_0_) for CD8^+^ T cells was 1.9-fold higher for Group 5 than Group 3 (only gp100-I on NPs) after gp100-I incubation. Both Group 4 (gp100-I on NPs mixed with gp100-II on NPs) and Group 5 showed high gp100-II-specific CD4^+^ T cell proliferation (Fig. 5F), but only the dual-antigen NP [Group 5] gave a statistically significant increase in gp100-I-specific CD8^+^ T cell proliferation compared to the single-antigen NPs (Groups 2 and 3) (Fig. 5E). Interestingly, Group 4, in which the NPs displayed either MHC class I or II peptide, showed antigen-specific T cell proliferation upon stimulation but demonstrated minimal IFN-γ and no IL-2 production beyond that of the Group 1 control. Previously published studies have posited that proliferation and cytokine production are not necessarily linked for antigen-specific T cells *in vitro* or *in vivo* [67,68]. While future studies would be needed to discern the mechanistic cause for the low cytokine production, the cells in Group 4 nevertheless proliferated and produced low amounts of IFN-γ in an antigen-specific manner compared to PBS-treated mice, indicating that they did perform effector functions and were not anergic. One possible explanation for the observation that these cells were not producing cytokines higher than the other NP groups is because they were in the process of proliferating, or had recently done so, as T cells cytokine production profiles can vary depending on time-dependent inflammatory state and T-cell subset [27, 69]. Another possible explanation is that IL-2 was consumed by the cells in Group 4 during T cell proliferation, which is consistent with autocrine signaling from these cells, where IL-2 is taken up by the cells that produced it [32]. This mechanism may also explain why Group 5 induced significantly higher IFN-γ production than Group 3 but not an increase in IL-2 levels. Altogether, the data in Fig. 5C–F suggest that immunization with MHC class I peptide drives Th1 cytokine production, while inclusion of MHC class II peptide promotes T-cell proliferation in Groups 4 and 5, possibly through autocrine or paracrine IL-2 signaling [32,70].

#### 3.2.3. Co-delivery of MHC class I and II gp100 epitopes on dual-antigen NPs significantly increased survival for mice with melanoma tumors

**Fig. 6.**
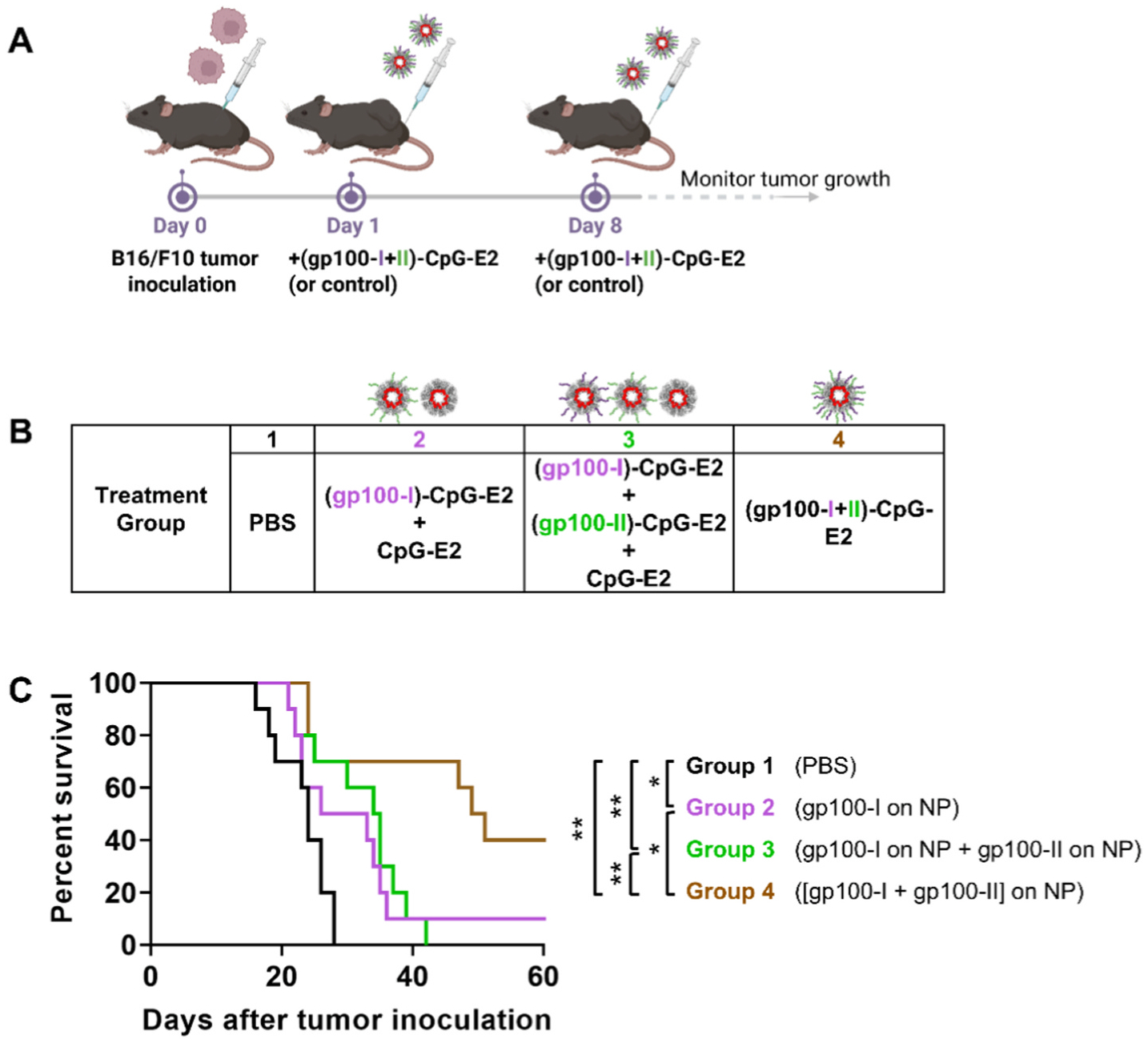
Melanoma treatment with E2 NPs co-conjugated with MHC class I and class II epitopes yielded the longest survival times. **(A)** Experimental schedule for tumor inoculation and treatment. **(B)** Summary of treatment groups. Compositions of these groups (NP, adjuvant, antigens) is described in Fig. 5B. **(C)** Overall survival of mice. Black, purple, green, and brown lines represent treatment by Groups 1, 2, 3, and 4, respectively, as described in (B). *N*=10. Statistics: log-rank test. *p ≤ 0.05, **p ≤ *0.01*.

We examined the potency of the (gp100-I + II)-CpG-E2 NPs to treat B16/F10 melanoma (Fig. 6). Groups in this tumor study included immunization with PBS [Group 1]; conventional NPs displaying gp100-I [Group 2]; a mixture of NPs individually displaying either gp100-I or gp100-II, though not on the same NP [Group 3]; and dual-conjugated NPs [Group 4]. Overall survival, tumor volumes, and percent changes in mouse body weight are presented in Fig. 6C, S4, and S5, respectively.

Our data show that mice immunized with the dual-antigen NPs [Group 4] out-performed all other groups to prolong survival (Fig. 6C). While 70% of mice in Group 4 survived past day 40, only 0%, 10%, and 10% survived for Groups 1, 2, and 3, respectively. Furthermore, 50% of mice with palpable tumors in Group 4 showed linear tumor growth from day 25 and onward, in contrast to all mice in Groups 1, 2, and 3, which experienced exponential tumor growth (Fig. S4). All mice treated with PBS developed terminally-sized tumors by day 30, and 40% of mice treated with a mixture of single-antigen NPs [Group 3] had terminal tumors by this time. Mice from Group 2 and Group 3 followed similar tumor growth trends over time (Fig. S4B–S4C). Therefore, our results demonstrate that the dual-antigen NP vaccine gave the highest survival numbers and the slowest average tumor growth rate, even out-performing Group 3, which consisted of the same individual components and doses, but delivered antigens in two different NPs. Early body weight changes were comparable across all groups after treatments (days 1 and 8), and the body weight of all nanoparticle-treated mice gradually increased over time with no observable adverse effects, supporting the safety of the vaccines (Fig. S5).

### 3.3. Nanoparticle vaccine investigations for the CT26 colon carcinoma model

Our studies with the B16/F10 melanoma model above demonstrated the advantage of incorporating an MHC class II epitope onto MHC class I peptide-decorated NPs to activate CD4^+^ T cells in support of the cytotoxic CD8^+^ T cells. To examine the extent of applicability of this strategy, we tested this approach in an alternative tumor model: CT26 colon carcinoma in its syngeneic mouse strain, BALB/c. The melanoma model used C57BL/6 mice, a strain biased towards Th1 responses [47]. Th1 cells secrete inflammatory cytokines (e.g., IFN-γ), which promote CD8^+^ T cell cytotoxicity and induce immune-mediated tumor cell death [71, 72]. In contrast, BALB/c mice are Th2-biased [73]. Th2 cells produce cytokines (e.g., IL-10) that suppress Th1 responses and T cell cytotoxicity while supporting B cells for humoral immunity [74]. Therefore, Th2 responses are associated with poor cancer prognoses [74], and anti-tumor immunity in the BALB/c model would likely be more difficult. Furthermore, Th1/Th2 cytokine biases have been observed in humans and are dependent upon many factors, including genetics, race, and psychological state, so evaluating therapeutics across a range of Th biases is important for clinical translation [51–53]. Therefore, we investigated whether NPs delivering an MHC class II epitope could also effectively treat CT26 colon carcinoma tumors in the BALB/c mouse model [75].

#### 3.3.1. Immunization with single-antigen CT-II NPs induced an antigen-specific Th1 response, even in Th2-biased BALB/c mice

**Fig. 7.**
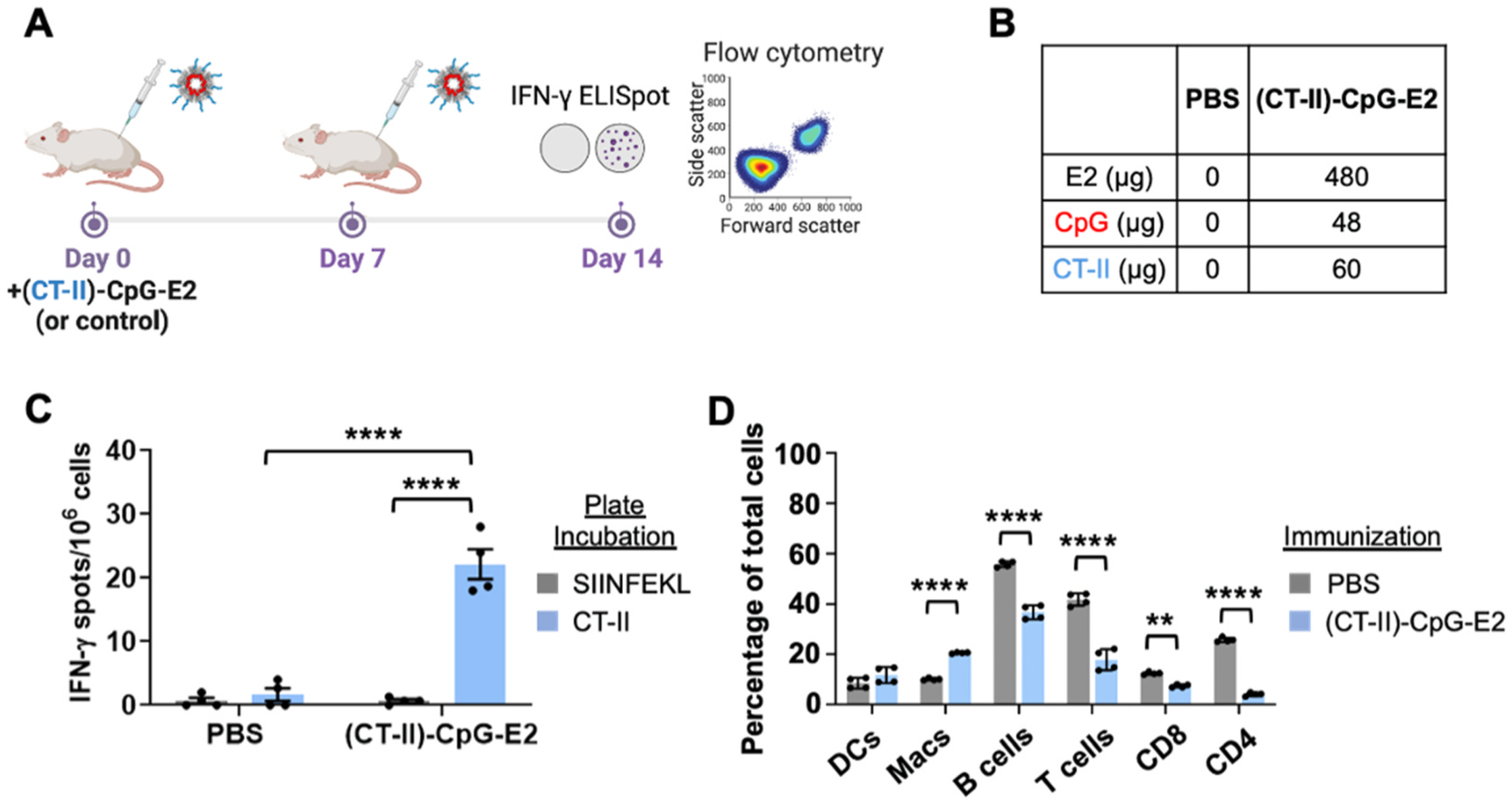
Immunization with E2 NPs displaying MHC class II antigen peptide from CT26 elicited antigen-specific IFN-γ responses and modulated immune populations. **(A)** Immunization schedule. **(B)** Nanoparticle vaccine components per immunization dose, including the amount of E2, CpG, and peptide. **(C)** IFN-γ response from splenocytes incubated with CT-II or an irrelevant peptide SIINFEKL (control) as determined via ELISpot. *Mean* ± *SEM. N*=*4. Statistics: Two-way ANOVA with post-hoc Bonferroni's test*. **(D)** Percentages of DCs, macrophages, B cells, T cells, CD8^+^ T cells, and CD4^+^ T cells from splenocytes after immunization with (CT-II)-CpG-E2 NPs or PBS. *Mean* ± *SD. N*=*4. Statistics: One-way ANOVA with post-hoc Bonferroni's test*. **p ≤ 0.01, ****p ≤ *0.0001*.

We immunized BALB/c mice with (CT-II)-CpG-E2 NPs to evaluate antigen-specific IFN-γ secretion, cytokines for Th1 responses, and changes in immune cell populations in secondary lymphoid tissue, potentially associated with immune activation (Fig. 7 and S6). The CT-II peptide was selected as the MHC II antigen (Fig. 2), based on its previously reported immunogenicity [10,76].

Splenocytes from mice immunized with (CT-II)-CpG-E2 showed a significantly higher CT-II-specific IFN-γ response compared to those from PBS-treated mice (13.3-fold difference in IFN-γ-producing cells), with negligible IFN-γ responses to the irrelevant SIINFEKL peptide (Fig. 7C). Furthermore, the resulting cell population trends of splenocytes were similar to those observed in the (gp100-II)-CpG-E2 immunization studies, including increased macrophage percentages and lower T cell percentages (both CD8^+^ and CD4^+^ T cells) (Fig. 7D). Average macrophage and T cell percentages in the draining LNs (Fig. S6) also exhibited similar trends to those observed in the spleen. Although DC percentages did not significantly increase, spleens, inguinal LNs, and axillary LNs collected from (CT-II)-CpG-E2 NP-immunized mice were larger than those from the PBS group (Fig. S2B, Groups G vs. A), indicating that more immune cells were present in these secondary lymphoid tissues after (CT-II)-CpG-E2 immunizations. Importantly, these (CT-II)-CpG-E2 NPs could induce a Th1 response, even in the BALB/c mice; therefore, this dosage of CT-II was used in the subsequent studies that included the MHC class I antigen.

#### 3.3.2. Co-delivery of MHC class I and II CT26 peptides on dual-antigen NPs induced greater antigen-specific Th1 responses in BALB/c mice

**Fig. 8.**
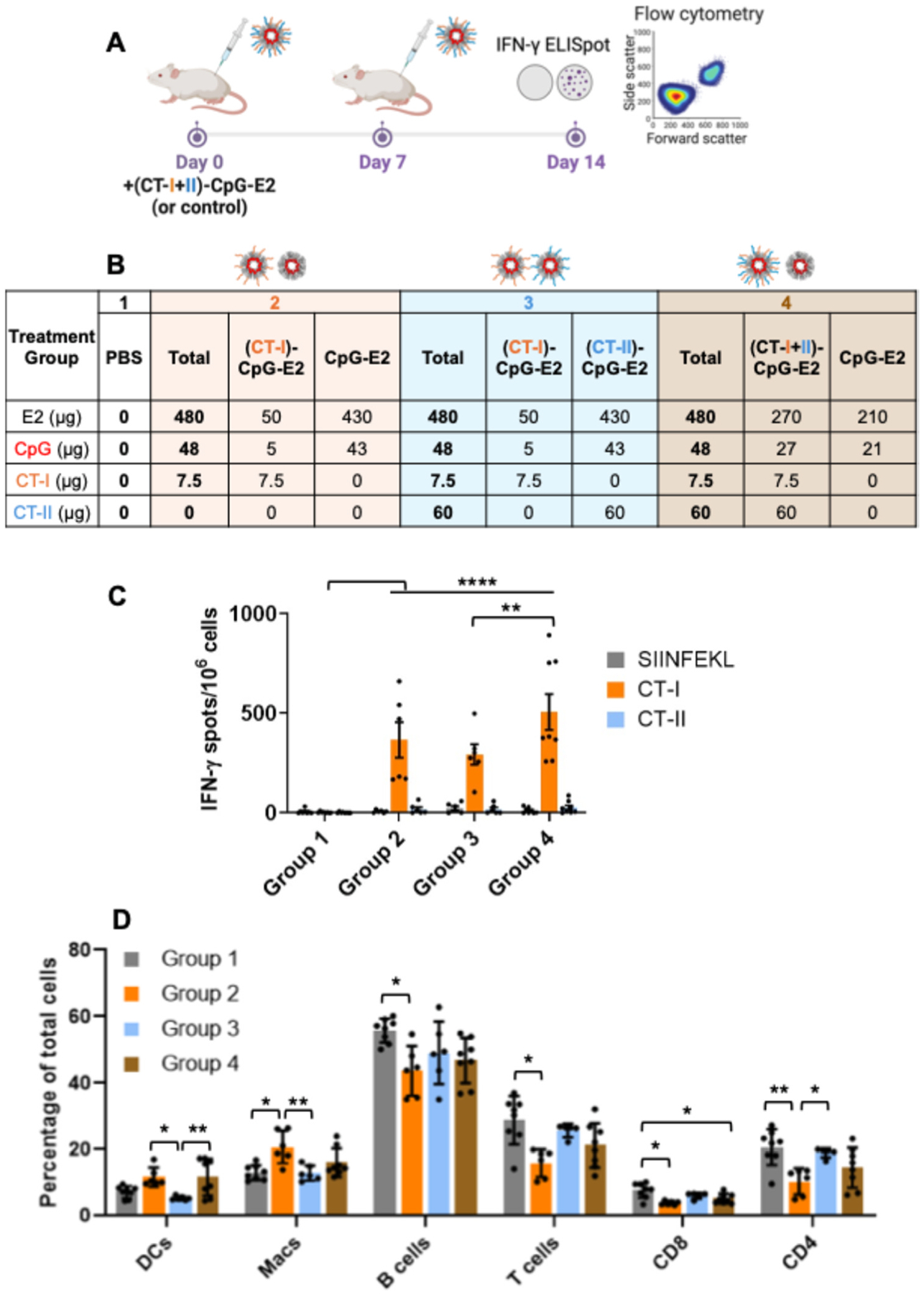
Immunization with E2 NPs co-delivering MHC class I and II colon carcinoma epitopes induced higher antigen-specific IFN-γ and modulated immune populations in the spleen. **(A)** Immunization schedule. **(B)** Nanoparticle vaccine components per immunization, including the amounts of E2, CpG, and peptides per dose. CpG-E2 was included in Groups 2 and 4 to ensure that all NPs groups receive equivalent E2 and CpG doses. **(C)** IFN-γ producing spots per million splenocytes incubated overnight with an irrelevant peptide (SIINFEKL), CT-I, or CT-II. *Mean* ± *SEM. N*≥6. Statistics: Two-way ANOVA with post-hoc Bonferroni's test. **p ≤ 0.01, ****p ≤ *0.0001*. **(D)** Flow cytometry staining analyses for DCs, macrophages, B cells, T cells, CD8^+^ T cells, and CD4^+^ T cells from splenocytes of immunized mice. *Mean* ± *SD. N*≥6. One-way ANOVA with post-hoc Bonferroni's test. *p ≤ 0.05, **p ≤ *0.01*.

To evaluate co-delivery of MHC class I and II epitopes by NPs to Th2-biased mice, animals were immunized with the following: PBS [Group 1], CT-I peptide on CpG-E2 NPs [Group 2], CT-I and CT-II on separate NPs [Group 3], and dual-antigen CT-I and CT-II NPs [Group 4] (Fig. 8A and B). To enable group-to-group comparisons, the CpG and E2 amounts were set equivalent across all NP groups by addition of CpG-E2 NPs to Groups 2 and 4. A 7.5-μg dose of the MHC class I peptide (CT-I) on NPs was selected for experiments, as this was sufficient to induce IFN-γ responses in mice (Fig. S7A), while not being a saturating dose based on DC numbers (Fig. S7B).

##### Effects of the MHC Class II peptide.

After immunization, the average number of CT-I-specific IFN-γ-producing splenocytes from the dual-antigen NPs [(CT-I + II)-CpG-E2; Group 4] was 1.4-times higher than the effects from the NPs with only MHC class I peptides [(CT-1)-CpG-E2; Group 2], as measured by ELISpot (Fig. 8C). Although this difference was not statistically significant, cytokine secretion analyses showed significantly higher concentrations of the Th1-related molecules IFN-γ (8.8-fold), TNF-α (2.9-fold), and IL-2 (2.6-fold), relative to immunization with CT-I-only NPs [Group 2] after stimulation with CT-I (Fig. S8). All NP groups also gave higher specific cytokine responses than the PBS control group. The data for IFN-γ, taken together, suggest that more IFN-γ was produced on average per cell in Group 4 than in Group 2.

Paracrine IL-2 production has been proposed as a mechanism for CD4^+^ T cells to augment CD8^+^ T cell expansion and effector differentiation [32]. Because mice immunized with single-antigen CT-I NPs [Group 2] or dual-antigen NPs [Group 4] received the same doses of CT-I, the significant increase in Th1 cytokine production following CT-I stimulation *ex vivo* (Fig. S8) is likely due to the incorporation of the MHC class II peptide for CD4^+^ T cell recognition. Therefore, the combined data in Fig. 8 and S8 strongly support that the dual-peptide NPs elicited a greater Th1 response than NPs with only the MHC class-I peptide, even in a Th2-biased mouse model.

Th2 cytokines (IL-4, IL-5, IL-10, and IL-13) were assessed but were below the detection limit for all groups. This could be attributed to the co-delivery of the Th1-skewing CpG adjuvant [77]. Although immunization with either (CT-I)-CpG-E2 or (CT-I + II)-CpG-E2 induced higher IL-6 secretion compared to the PBS controls, there was no significant difference between these NP immunization groups (Fig. S8D). IL-6 is a pleotropic cytokine that can support Th2 responses, but it also exerts pro-inflammatory effects, promotes cytotoxic T cell differentiation, and suppresses regulatory T cell differentiation, which are favorable outcomes for anti-tumor vaccine efficacy [78,79]. Thus, the attachment of CT-II peptides onto (CT-I)-CpG-E2 NPs did not increase Th2 cytokine production compared to (CT-I)-CpG-E2 alone; this was a desirable result, given that Th2 responses can suppress anti-tumor activity.

##### Effects of antigen localization on the same vs. different NPs.

Our data show that immunization with the dual-antigen NPs [(CT-I + II)-CpG-E2; Group 4] led to a 1.7-fold increase in CT-I-specific IFN-γ-producing cells, compared to the mixture of the two single-antigen NPs [(CT-I)-CpG-E2 + (CT-II)-CpG-E2; Group 3] with p < 0.01 (Fig. 8C) and a significantly higher percentage of DCs in the spleen (Fig. 8D). Additionally, Group 4 exhibited a lower average percentage of T cells (including all T cells and their CD4^+^ and CD8^+^ subsets) than Group 3, though differences were not statistically significant. Thus, co-delivery of the MHC class I and II antigens on a single particle elicited higher immune responses than mixtures of the single-antigen particles.

#### 3.3.3. Co-delivery of MHC class I and II CT26 peptides on dual-antigen NPs significantly increased survival for mice with colon carcinoma

**Fig. 9.**
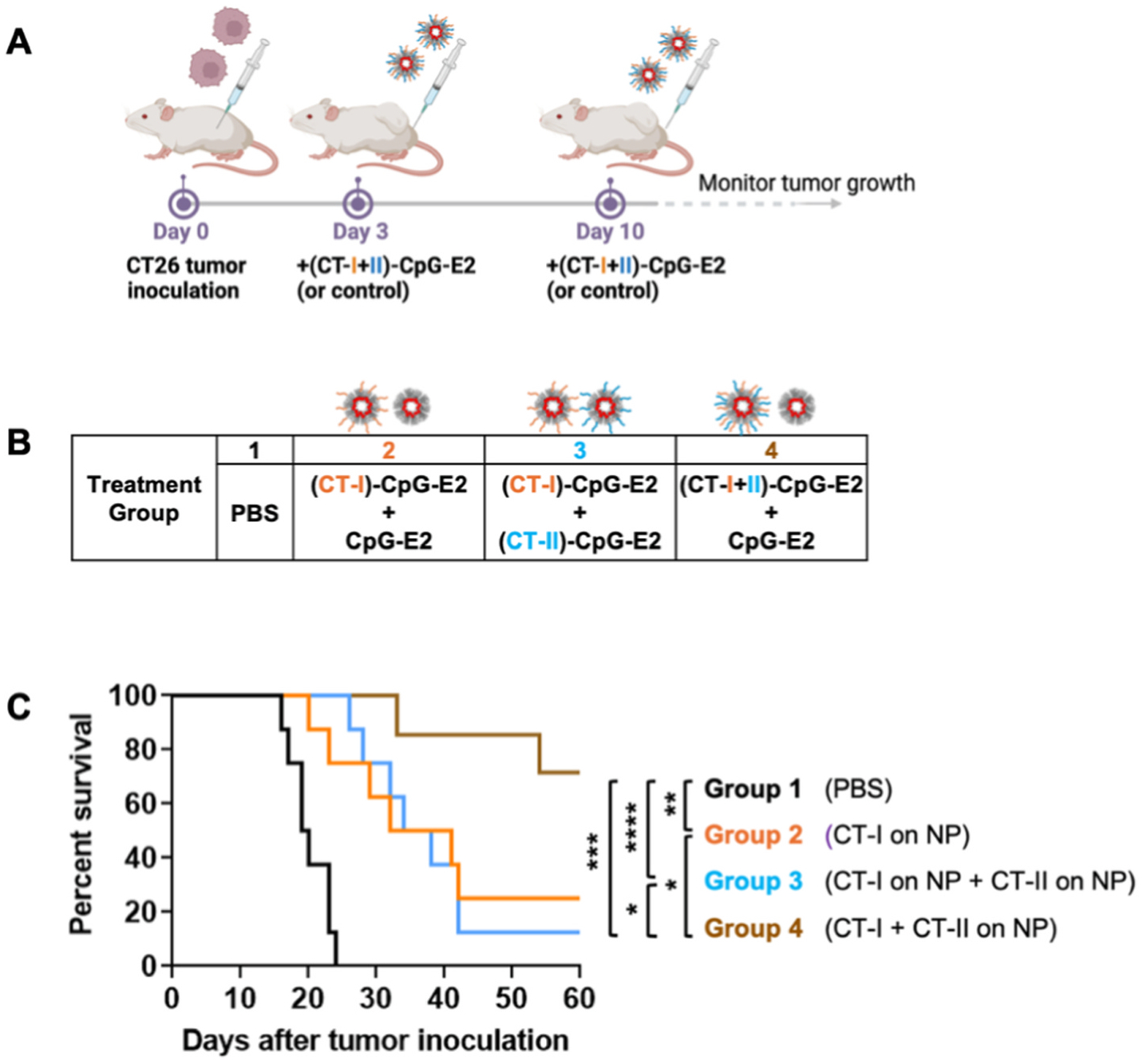
Colon carcinoma treatment with E2 NPs co-conjugated with MHC class I and class II epitopes most significantly increased survival. **(A)** Experimental schedule for tumor inoculation and treatment. CT26 cells were inoculated on day 0, and treatments were administered on days 3 and 10. **(B)** Summary of treatment groups. Composition of the treatment groups (NP, adjuvant, antigens) is described in Fig. 8B. **(C)** Overall survival of mice. Black, orange, blue, and brown lines represent treatments by Groups 1, 2, 3, and 4, respectively, as described in panel (B). *N*≥*7*. Statistics: log-rank test. *p ≤ 0.05, **p ≤ 0.01, ***p ≤ 0.001, ****p ≤ *0.0001*.

To test colon carcinoma treatment efficacy, BALB/c mice were inoculated with CT26 cells, immunized with NPs or PBS control, and evaluated for tumor growth and overall survival (Fig. 9A). Groups included PBS as vehicle control [Group 1], single-antigen NPs delivering CT-I [Group 2], a mixture of single-antigen NPs delivering CT-I and CT-II on separate NPs [Group 3], and dual-antigen NPs co-delivering CT-I and CT-II [Group 4] (Fig. 9B). As before, CpG-E2 was included in NP formulations to match the amounts of E2 scaffold and CpG adjuvant between the groups.

All NP-treated groups [Groups 2–4] exhibited significantly improved survival rates compared to the PBS control [Group 1], with co-conjugated NP immunization [Group 4] yielding a significantly higher survival percentage than all other groups (Fig. 9C and S9). On day 60, ~71% of mice in Group 4 survived, compared to only 25% of mice treated with NPs with single-antigen, CT-I only [Group 2] or ~13% for mice treated with a mixture of single-antigen NPs [Group 3]. No statistically significant difference in survival rates was observed between Groups 2 and 3. As with the melanoma model (Fig. S6), all mice receiving NPs increased in body weight over time (Fig. S10). These data support the hypothesis that MHC class I and class II antigens should be co-delivered on the same NPs for greater efficacy.

## 4. Discussion

### 4.1. The delivery of MHC class II antigen on NPs improved antigen-specific Th1 responses

Although NP-based cancer vaccines conventionally incorporate solely MHC class I antigens [6,80,81], our studies here also support the use of MHC class II peptides (gp100-II, CT-II) on NPs to improve anti-tumor efficacy. We observed antigen-specific proliferation of splenocytes from mice immunized with gp100-II on NPs (Fig. 4C). In both tumor models, cell population analysis of the splenocytes and LN cells also suggested the activation of T cells (Figs. 4D–E, 7D, and S6); with increased DC trafficking to LNs, the decrease in percentages of T cells in lymphoid organs could suggest T cell egressing from the lymph organ to the periphery [82]. Consistent with this observation, our prior studies and Fan *et al*. [83] demonstrated that despite the lack of an increase in T cell frequency after immunization, T cells exhibited increased antigen-specific proliferation, enhanced cytolytic activity, and demonstrated effective tumor protection [35]. In our current study, a higher number of IFN-γ producing splenocytes resulted from CT-II stimulation in the BALB/c model (Fig. 7C), indicating enhanced antigen-specific T cell activation and Th1 response following NP immunization despite using this Th2-biased model [27]. All of these effects are likely facilitated by DCs and CD4^+^ T cells [82]. Our data lend additional support to the accumulating evidence that CD4 T cell responses alone can play an important role in anti-tumor vaccine immune responses and efficacy [10,11,65,66,76], and they demonstrate the advantage of including an MHC class II epitope in NP-based cancer vaccines to induce T cell proliferation and elicit an antigen-specific IFN-γ response, even in a Th2-biased murine model.

### 4.2. Dual-antigen NPs induced greater antigen-specific Th1 responses than mixtures of dose-equivalent, single-antigen NPs

In both the melanoma and colon carcinoma models, immunization with dual-antigen NPs yielded increased MHC class I-specific Th1 cytokine responses (e.g., IFN-γ, IL-2, TNF-α) compared to the single-antigen NP mixtures or MHC class I peptide-only NPs. This indicated that the co-delivery of MHC class I and II antigens on the same NP, and hence likely to the same DC (e.g., Fig. 1, C), improved cell-mediated Th1 responses (Figs. 5C–D, 8C, and S8A–C). We consistently observed that IFN-γ immune responses (Figs. 5C and 8C) from MHC class I and II antigens on separate, single-antigen NPs were not enhanced compared to MHC class I peptide-only NPs (Fig. 1A and B). A possible explanation is that in the mixture of single-antigen NPs, a large proportion of NPs displayed only MHC class II antigens due to higher concentrations in the dosages. Although the total antigen, adjuvant, and NP doses were matched across groups, controlling the peptide-to-NP ratio was challenging. Therefore, the mixture may have reduced the probability of the same DC simultaneously activating both CD4^+^ and CD8^+^ T cells, causing a diluted education towards MHC class I antigen. Alternatively, our data speak to potential spatial and temporal constraints for optimal DC activation of T cells (Fig. 1, C), in addition to the recently appreciated spatial factors in the tumor microenvironment [84–87]. Furthermore, in the colon carcinoma model, separately delivered antigen NPs [Group 3] did not induce an increased DC population in the spleen compared to co-conjugated NPs [Group 4] or single-antigen NPs delivering only MHC class I antigen [Group 2] (Fig. 8D). This could be explained by the single-antigen NPs delivering only MHC class II antigen also failing to increase splenic DC populations, and that Group 3 contained 8.6-fold more MHC class II-bearing NPs than MHC class I-bearing NPs (Figs. 7D and 8D). Possible explanations for the lack of increased DC percentages following MHC class II NP vaccination are the lower immunogenicity of MHC class II peptides compared to MHC class I peptides [10,88], or that immune activation in response to MHC class II may be centered primarily in the draining LNs rather than the spleen. Higher MHC class II antigen-specific IL-2 cytokine secretion was also observed following immunization with dual-antigen (gp100-I + II)-CpG-E2 and (CT-I + II)-CpG-E2 NPs (Fig. 5D and S8C). IL-2 can support T cell proliferation, expression of effector molecules, and pro-inflammatory cytokine secretion [27], so these data suggest the importance of CD4^+^ T cell support of CD8^+^ T cells for the dual-antigen vaccines. The dual-antigen (gp100-I + II)-CpG-E2 NPs also induced the greatest antigen-specific CD8^+^ T cell proliferation and the second-highest CD4^+^ T cell proliferation (Fig. 5E and F). The increased CD8^+^ T cell proliferation observed with dual-antigen NPs compared with separately delivered antigens is consistent with findings from Teplensky *et al*., who reported a two-fold increase in CD8^+^ T cell proliferation towards ovalbumin peptides [89].

Notably, a Th2 cytokine response, which can be pro-tumor [48], was not observed following immunization with (CT-I + II)-CpG-E2 NPs, despite the use of CT-II peptide and Th2-biased BALB/c mice; this could be the effect of using CpG, which is a Th1-inducing adjuvant [77]. Our findings are similar to those of Salomon *et al*., where CD4^+^ T cells from mice immunized three times with an RNA vaccine for CT-II peptides (together with four other MHC class I and II epitopes) exhibited a Th1 phenotype, with the dominant secretion of Th1 or pro-inflammatory cytokines, such as IFN-γ, TNF-α, IL-2, and IL-1β [11].

Regardless of tumor model, our data support our hypotheses for the mechanistic advantages of delivering both MHC classes of antigens to the same DC, as well as the benefits of delivering both MHC class I and II antigens by NP vaccines to enhance antigen-specific Th1 cytokine secretion and promote greater CD4^+^ or CD8^+^ T cell proliferation (Fig. 1C). While other immunological studies did not focus on the design of NPs specifically, our observations here are consistent with previous studies evaluating the interplay between DCs, CD4^+^ T cells, and CD8^+^ T cells. The heightened local IL-2 levels around a DC recruiting both CD4^+^ and CD8^+^ T cells simultaneously can enhance responses by both T helper and cytotoxic T cells [29]. Other studies have reported improved CD8^+^ T cell recruitment and IFN-γ secretion when activated DCs displayed peptides by both classes of MHC, rather than only MHC I [25,31]. Therefore, direct ternary interaction between a DC, CD4^+^ T cells, and CD8^+^ T cells may be critical for improved Th1 and proliferation responses [27]. Together with the findings from MHC class II peptide-only NP immunizations, these results suggested that while MHC class I single-antigen NPs can produce robust anti-tumor immune responses, the co-delivery of both MHC class I and II antigens on the same NPs can elevate targeted cytotoxic T cell responses.

### 4.3. The co-delivery of MHC class I and II antigens on NPs improved melanoma and colon carcinoma treatment efficacy

For both models, our data demonstrated that immunization with the dual-antigen NPs improved tumor treatment efficacy compared to other NP groups. Melanoma or colon carcinoma treatment with the dual-antigen NPs [Group 4] was significantly more efficacious than treatment with single-antigen NPs delivering only MHC class I antigen [Group 2] or both MHC class I and II antigens on separate vehicles [Group 3] (Figs. 6C and 9C). Furthermore, in both tumor models, there was no significant difference in survival between Groups 2 and 3, showing that the MHC class II peptides, when they are delivered on a separate NP, did not improve efficacy or contribute to an additive effect. In other words, immunizations using MHC class II antigen improved survival only when co-delivered with MHC class I antigen on the same NPs.

While it is established that CD4^+^ T cells contribute to cytotoxic activity through mechanisms such as secretion of dendritic cell-stimulating cytokines and enhancement of antigen presentation via CD40 activation [90,91], Espinosa-Carrasco *et al*. [92] has reported the advantages of immune triad formation, where a single DC interacts with both CD8^+^ and CD4^+^ T cells, as proposed in Fig. 1, Scenario C. These immune triads were shown to play an important role in licensing CD8^+^ T cell cytotoxicity and facilitating cancer cell elimination. However, previous studies did not perform comparisons between the spatial configuration of different NP formulations in a tumor treatment setting [65,66]. Nevertheless, we believe that the combination of MHC class I and MHC class II peptides delivered on the same nanoparticle, along with a potent adjuvant CpG, allows for the formation of these immune triads and promotes a more robust and durable anti-tumor immune response. Our studies here systematically examined comparisons between the mixtures of MHC class I/II single-antigen NPs and dual-antigen NPs in tumor treatment settings and have identified an important consideration in the design of peptide-delivering anti-cancer vaccine therapies.

Compared to other melanoma or colon carcinoma NP therapies, our dual-peptide NPs were relatively effective for their component dosages and administration schedule. Melanoma treatment with (gp100-I + II)-CpG-E2 NPs utilized much lower gp100 antigen doses (5–15 μg versus ~100 μg in prior studies) [65,66], implemented a two-dose rather than a three- or four-dose NP treatment schedule [66,93], and did not require combination with additional strategies, such as with checkpoint immunotherapy (e.g., anti-PD1) [6] or DC-targeting ligands (e.g., mannose) [65,66]. Other protein-based NP platforms complexed with gp100 proteins caused earlier onset and development of larger tumors within 30 days [17,94], compared to our two-dose treatment with (gp100-I + II)-CpG-E2 NPs (Fig. S4D).

Treatment of colon carcinoma with DC-based or lipid nanoparticle (LNP)-based vaccines comprising both MHC class I and II antigen peptides have also been explored. However, these studies did not specifically investigate the importance of delivering MHC class I and II peptides on the same NP and differ from ours in several aspects [10,11,83,95]. The prior cancer vaccine studies incorporated up to 20 total MHC class I and II RNA epitopes and required up to seven doses of LNPs, additional antibody treatments, or local radiotherapy to achieve effective tumor treatment [10,11,83,95]. Nevertheless, the growing number of studies investigating vaccines delivering MHC class I and II antigens highlights the increasing interest in activating both CD8^+^ and CD4^+^ T cells simultaneously.

Our studies strongly support that both MHC classes of antigens should be co-delivered within the same NP vaccine vehicles, rather than on separate NPs or using MHC class I antigen alone (without MHC class II antigen), for significantly improved potency in the treatment of tumors. The consistency of our results across two tumor models, despite differing inherent Th biases in the mouse strains [49,96], reinforces the broad applicability of designing NP immunotherapies to educate DCs for the simultaneous activation of CD8^+^ and CD4^+^ T cells, and supports the DC presentation scheme suggested in Scenario C of Fig. 1. Our investigation offers a more effective NP-based strategy for immune system education and potential cancer treatment than the conventional method of delivering only MHC class I epitopes.

## 5. Conclusions

In these studies, we examined whether designing NP peptide vaccines to co-deliver or separately deliver MHC class I and II antigens would more effectively treat cancer. Both melanoma and colon carcinoma models were used to evaluate the breadth of efficacy for these designed NPs, which represented a range of MHC haplotypes and Th-biases, similar to variations observed in patient populations. Regardless of the tumor model, immunization with dual-antigen NPs, which enabled the co-delivery of MHC class I and II epitopes to the same DC (Fig. 1, C), resulted in enhanced antigen-specific secretion of Th1 or pro-inflammatory cytokines, such as IFN-γ.

Tumor treatment studies were performed to determine the functional efficacy of the co-delivery design. In both tumor models, the long-term survival of mice bearing tumors was significantly enhanced following treatment with dual-antigen NPs over all other groups, confirming that the trends we observed in immunization studies remained consistent in a tumor treatment setting. Furthermore, most mice treated with co-conjugated NPs exhibited a delay in exponential tumor growth by multiple weeks. The robustness of these trends is supported by consistency between immunizations and treatments in either the Th1-biased C57BL/6 or Th2-biased BALB/c mouse models. This work strongly supports that cancer NP immunotherapies should be designed to take advantage of DCs’ abilities to simultaneously activate both CD8^+^ T cells and CD4^+^ T cells through the co-delivery of MHC I and II antigens in the same NP delivery vehicle.

## Supplementary Material

1

## Data Availability

Data will be made available on request.

## Appendix A. Supplementary data

Supplementary data to this article can be found online at https://doi.org/10.1016/j.biomaterials.2026.124128.
