# Supplementary material for: Nanoparticle-mediated dual delivery of MHC class I and II antigens enhances T cell immunity and anti-tumor potency
Source: Biomaterials. Author manuscript; Available in PMC 2026 Jun 9. (PMC13249364; doi:10.1016/j.biomaterials.2026.124128)
Supplement: 1 [file NIHMS2180144-supplement-1.pdf]

Supplementary Information

Nanoparticle-mediated dual delivery of MHC class I and II antigens enhances T cell immunity and anti-tumor potency

Enya Li [§,1], Nina Butkovich [§,1], Jo A. Tucker [2,3],

Edward L. Nelson [2,3,5], Szu-Wen Wang [1,3,4,5,*]

Department of Chemical and Biomolecular Engineering [1]

Department of Medicine [2]

Chao Family Comprehensive Cancer Center [3]

Department of Biomedical Engineering [4]

Institute for Immunology [5]

University of California, Irvine, CA 92697, USA

[§] E. Li and N. Butkovich contributed equally to the manuscript and are co-first authors

* Corresponding author: S.-W. Wang (wangsw@uci.edu)

Supplementary Table

A

Nanoparticle	Mixed molar ratio			Molar ratio achieved		
	E2 monomer	MHC class I antigen	MHC class II antigen	E2 monomer	MHC class I antigen	MHC class II antigen
(gp100-I)-CpG-E2	1	10	0	1	3.6 ± 1.8	0
(gp100-II)-CpG-E2	1	0	10	1	0	2.1 ± 0.5
(gp100-I+II)-CpG-E2	1	1	2	1	0.7 ± 0.2	1.2 ± 0.3
(CT-I)-CpG-E2	1	10	0	1	3.5 ± 0.1	0
(CT-II)-CpG-E2	1	0	2	1	0	1.4 ± 0.1
(CT-I+II)-CpG-E2	1	1	3.5	1	0.7 ± 0.04	1.9 ± 0.2

B

Nanoparticle	Mixed mass ratio			Mass ratio achieved		
	E2 monomer	MHC class I antigen	MHC class II antigen	E2 monomer	MHC class I antigen	MHC class II antigen
(gp100-I)-CpG-E2	1	0.45	0	1	0.16 ± 0.081	0
(gp100-II)-CpG-E2	1	0	0.72	1	0	0.15 ± 0.036
(gp100-I+II)-CpG-E2	1	0.041	0.068	1	0.031 ± 0.0085	0.088 ± 0.024
(CT-I)-CpG-E2	1	0.43	0	1	0.15 ± 0.0047	0
(CT-II)-CpG-E2	1	0	0.21	1	0	0.14 ± 0.010
(CT-I+II)-CpG-E2	1	0.043	0.10	1	0.028 ± 0.0019	0.19 ± 0.016

Table S1. Conjugation ratios of components to E2 NP for each vaccine design. (A) Molar or **(B)** mass ratios of components (including gp100 or CT peptide antigens) to E2 monomer. The "mixed molar ratio" indicates the ratio added into the reaction mixture to achieve the final conjugation ratio ("molar ratio achieved"), per monomer. Each E2 NP is composed of 60 identical subunit monomers; therefore, the average antigen-to-NP ratio can be estimated by multiplying the antigen-to-monomer ratio by 60. *Mean ± SD. N≥3.*

Supplementary Figures

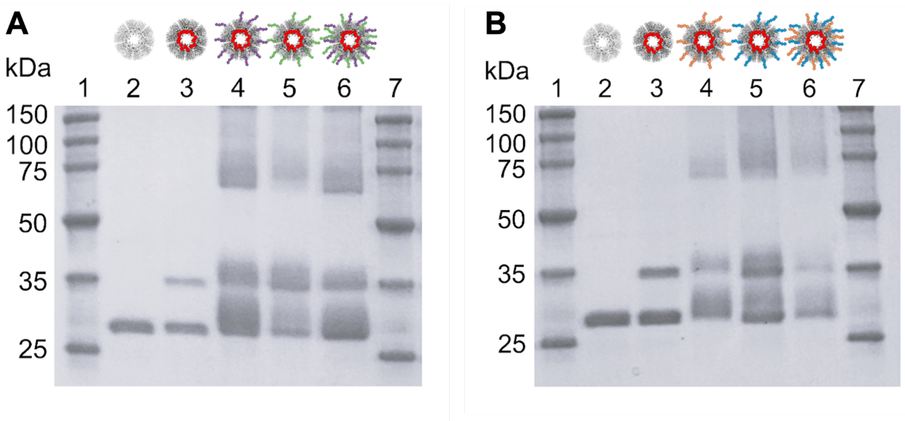

Figure S1. SDS-PAGE gel analysis confirmed conjugation of CpG and peptides. SDS-PAGE gels of E2 NPs as **(A)** melanoma or **(B)** colon carcinoma immunotherapeutic or controls. **(A)** Lanes: (1, 7) molecular weight ladder; (2) E2; (3) CpG-E2; (4) (gp100-I)-CpG-E2; (5) (gp100-II)-CpG-E2; and (6) (gp100-I+II)-CpG-E2. **(B)** Lanes: (1, 7) molecular weight ladder; (2) E2; (3) CpG-E2; (4) (CT-I)-CpG-E2; (5) (CT-II)-CpG-E2; and (6) (CT-I+II)-CpG-E2. The conjugation of CpG or peptides onto E2 monomers is supported by the shift in bands.

A

Group	A	B	C	D	E	F
Immunization	PBS	(gp100-II)-CpG-E2	(gp100-I)-CpG-E2	(gp100-I)-CpG-E2 + CpG-E2	(gp100-I)-CpG-E2 + (gp100-II)-CpG-E2 + CpG-E2	(gp100-I+II)-CpG-E2
E2 (μg)	0	110 (3.9 nmol)	50 (1.8 nmol)	180 (6.4 nmol)	180 (6.4 nmol)	180 (6.4 nmol)
CpG (μg)	0	11 (1.7 nmol)	5 (0.8 nmol)	18 (2.7 nmol)	18 (2.7 nmol)	18 (2.7 nmol)
gp100-I (μg)	0	0	5 (4.3 nmol)	5 (4.3 nmol)	5 (4.3 nmol)	5 (4.3 nmol)
gp100-II (μg)	0	15 (8.3 nmol)	0	0	15 (8.3 nmol)	15 (8.3 nmol)

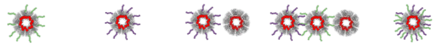

Group	G	H	I	J
Immunization	(CT-II)-CpG-E2	(CT-I)-CpG-E2 + CpG-E2	(CT-I)-CpG-E2 + (CT-II)-CpG-E2	(CT-I+II)-CpG-E2 + CpG-E2
E2 (μg)	480 (15 nmol)	480 (15 nmol)	480 (15 nmol)	480 (15 nmol)
CpG (μg)	48 (7.3 nmol)	48 (7.3 nmol)	48 (7.3 nmol)	48 (7.3 nmol)
CT-I (μg)	0	7.5 (6.2 nmol)	7.5 (6.2 nmol)	7.5 (6.2 nmol)
CT-II (μg)	60 (21 nmol)	0	60 (21 nmol)	60 (21 nmol)

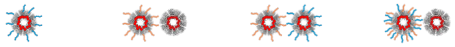

B

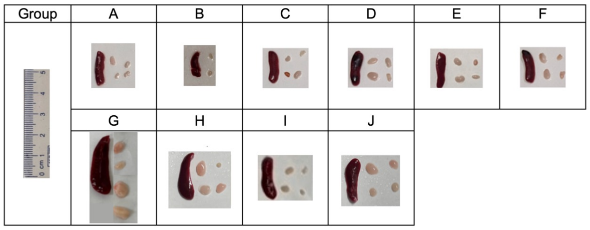

C

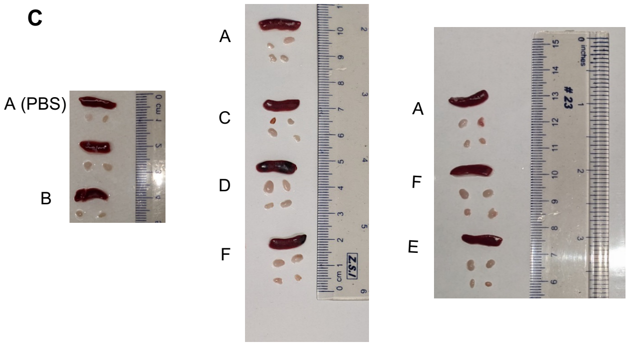

D

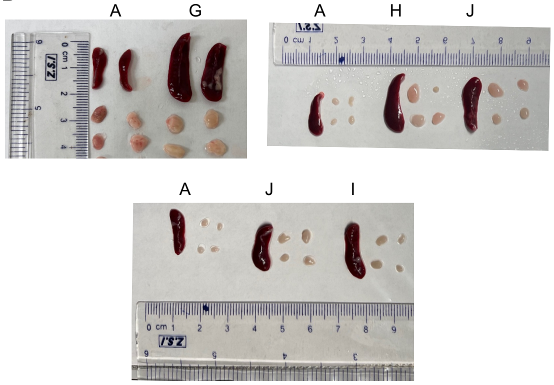

Figure S2. Immunization doses with nanoparticle vaccines delivering gp100 or CT antigens and resulting spleens and lymph nodes (LNs) after immunization. (A) Nanoparticle vaccine

components per immunization, including amount of E2, CpG, and peptide per dose (in both mass and molar amounts). **(B)** Representative images of spleens and LNs (axillary and inguinal) harvested on day 14 following immunization on day 0 and day 7 with conditions described in the table. All images are to scale with the rulers. **(C)** Representative original images of spleens and LNs from the gp100 antigen experimental groups (Groups A-F). **(D)** Representative original images of spleens and LNs from the CT antigen experimental groups (Groups A, G-J).

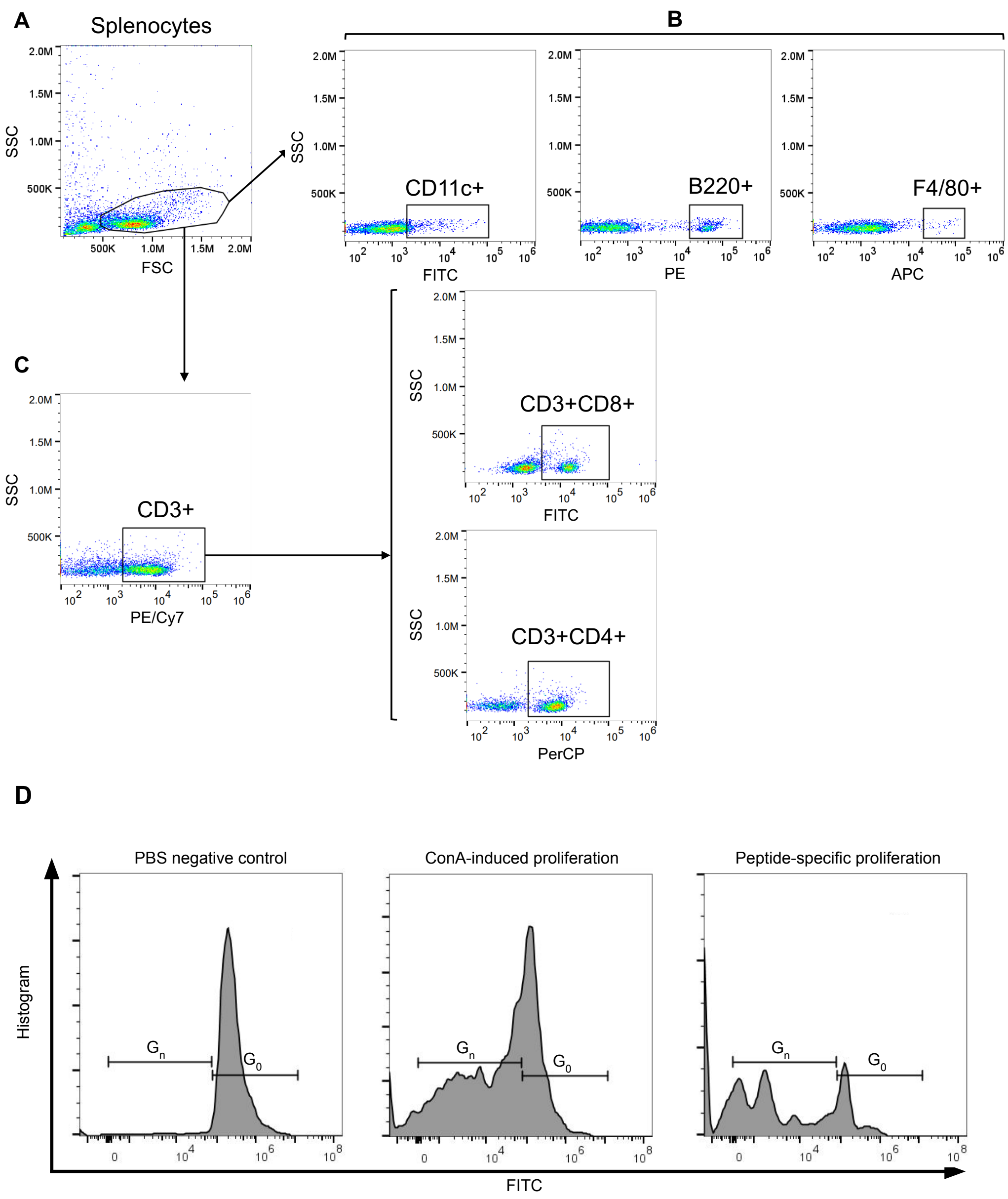

Figure S3. Flow cytometry analysis of splenocytes and representative histograms of proliferating and non-proliferating splenocytes stained with CFSE. Shown are representative scatter plots throughout the gating process. **(A)** All splenocytes were size-gated, followed by characterizing **(B)** antigen-presenting cell or **(C)** T cell markers. **(D)** Shown are histograms of gated cells which had been incubated for 72 hr with PBS (negative control), ConA (positive control), or recall peptide. FITC signal correlating to CFSE content is shown along the horizontal axes. While populations G_0 represent non-proliferated cells, daughter generations are highlighted by G_n. Minimal numbers of cells within daughter generations were present following PBS incubation, while a significant amount of proliferation resulted following stimulation with ConA.
SSC: side scatter. FSC: forward scatter. ConA: Concanavalin A.

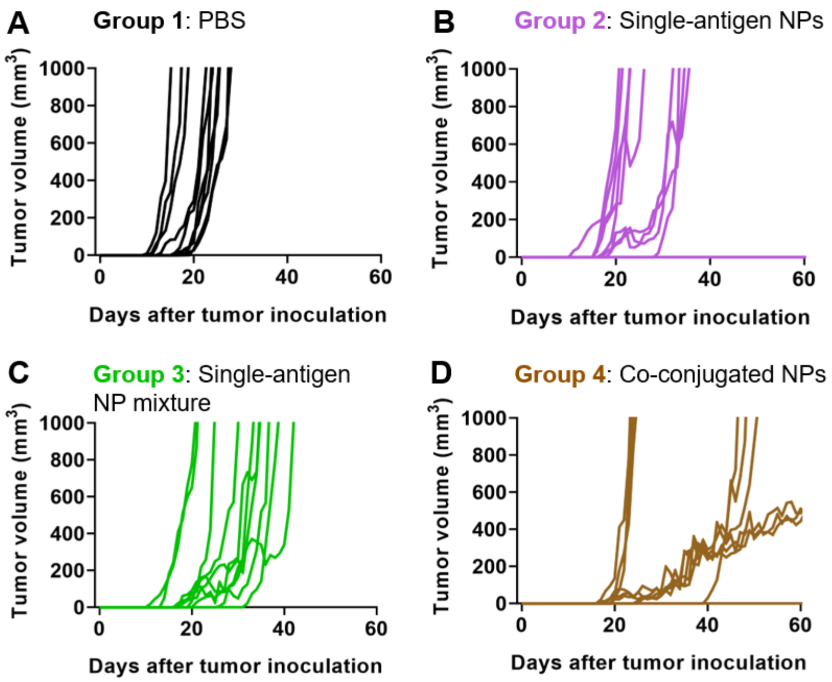

Figure S4. Treatment with E2 NPs decorated with MHC class I and II antigens delayed melanoma tumor growth. Tumor volumes of individual mice bearing melanoma over time. Tumor growth was monitored over several weeks following initial tumor inoculation on day 0 for mice treated on days 1 and 8 with **(A)** PBS, **(B)** (gp100-I)-CpG-E2 + CpG-E2, **(C)** (gp100-I)-CpG-E2 + (gp100-II)-CpG-E2 + CpG-E2, and **(D)** (gp100-I+II)-CpG-E2. Of the 4 groups, Groups 2 and 3 included treatments with single-antigen NPs, while Group 4 used NPs co-conjugated with both MHC class I and II epitopes. *N=10 biological replicates.*

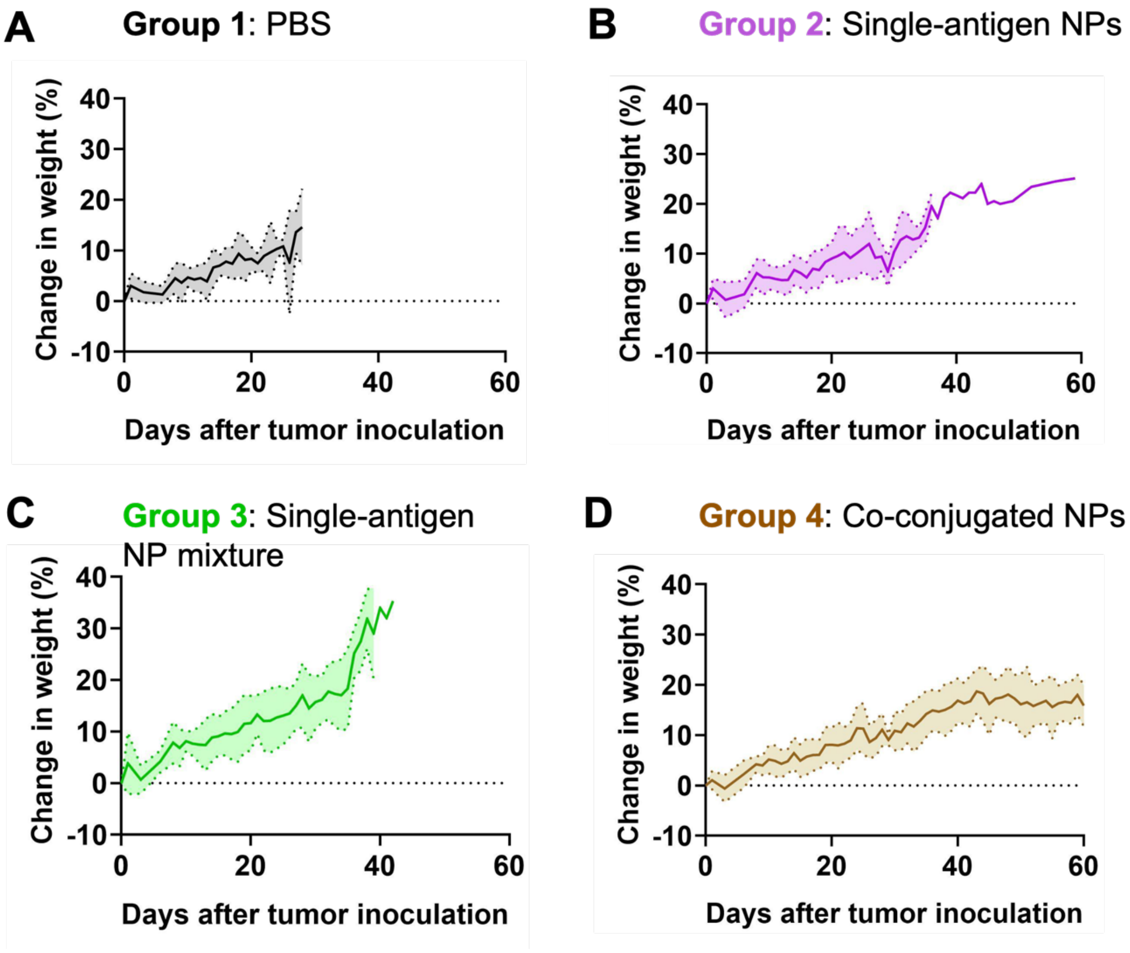

Figure S5. Mouse body weight over time. Shown are the percent changes in mouse body weight through day 60 after tumor inoculation, calculated relative to day 0 for each mouse. Treatment groups were: **(A)** PBS, **(B)** (gp100-I)-CpG-E2 + CpG-E2, **(C)** (gp100-I)-CpG-E2 + (gp100-II)-CpG-E2 + CpG-E2, and **(D)** (gp100-I+II)-CpG-E2. The dashed horizontal line (y = 0) represents no change in body weight relative to day 0. The shaded area represents the *mean* ± *SD. N=10 biological replicates.*

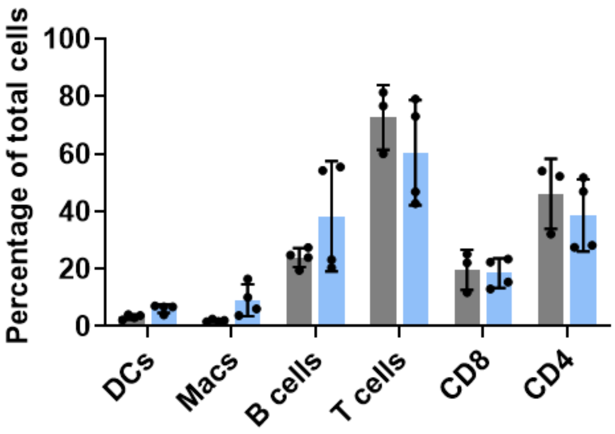

Figure S6. Immune populations in the LNs following immunization with E2 NPs displaying MHC class II colon carcinoma. Flow cytometry staining analyses for DCs, macrophages, B cells, T cells, CD8+ T cells, and CD4+ T cells from LNs. Gray and blue bars represent immunization with PBS or (CT-II)-CpG-E2, respectively. *Mean ± SD. N≥3.*

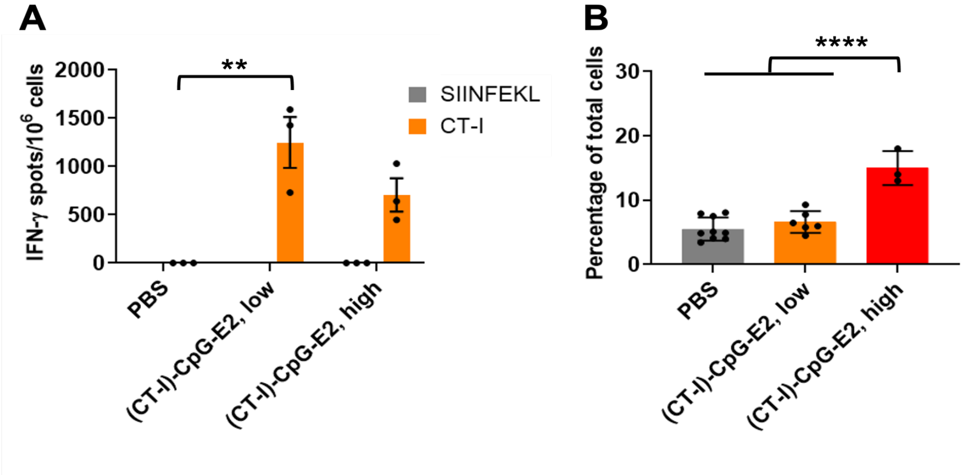

Figure S7. Immunization with differing doses of CT-I on NPs caused Th1 or DC responses in the spleen. We immunized mice on days 0 and 7 with PBS, a low dose of (CT-I)-CpG-E2 NPs, or a high dose of (CT-I)-CpG-E2 NPs and then performed IFN-γ ELISpot and assessed DC populations on day 14. Low and high doses of NPs contained 7.5 μg and 30 μg of CT-I, respectively. **(A)** IFN-γ producing spots per million splenocytes after overnight incubation with an irrelevant peptide (SIINFEKL) or CT-I. *Mean ± SEM. N=3. Statistics: One-way ANOVA with post-hoc Bonferroni's test. **p ≤ 0.01; p = 0.067 between high dose and PBS groups following CT-I incubation*. **(B)** Dendritic cell population in the spleen following immunization with a low or high dose of NPs. *Mean ± SD. N≥3. Statistics: One-way ANOVA with post-hoc Bonferroni's test. ****p ≤ 0.0001*.

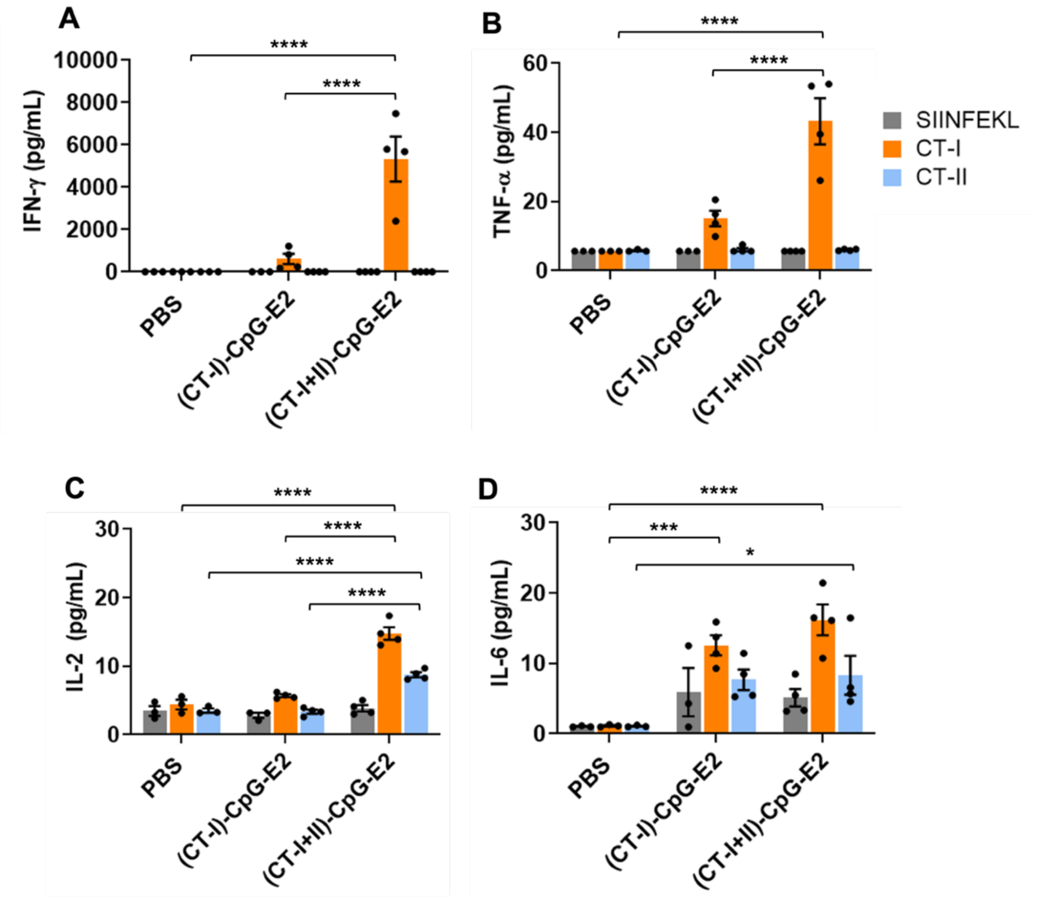

Figure S8. Immunization with E2 NPs co-delivering MHC class I and class II colon carcinoma antigens caused the greatest increase in antigen-specific IFN-γ, TNF-α, IL-2, and IL-6 cytokine production. (A) IFN-γ, **(B)** TNF-α, **(C)** IL-2, and **(D)** IL-6 concentrations as determined by LEGENDplex of conditioned media of splenocytes incubated with peptide. Gray, orange, and blue bars represent SIINFEKL (irrelevant peptide), CT-I, or CT-II incubation conditions, respectively. *Mean ± SEM. N≥3. Statistics: Two-way ANOVA with post-hoc Bonferroni's test. *p ≤ 0.05, ***p ≤ 0.001, ****p ≤ 0.0001.*

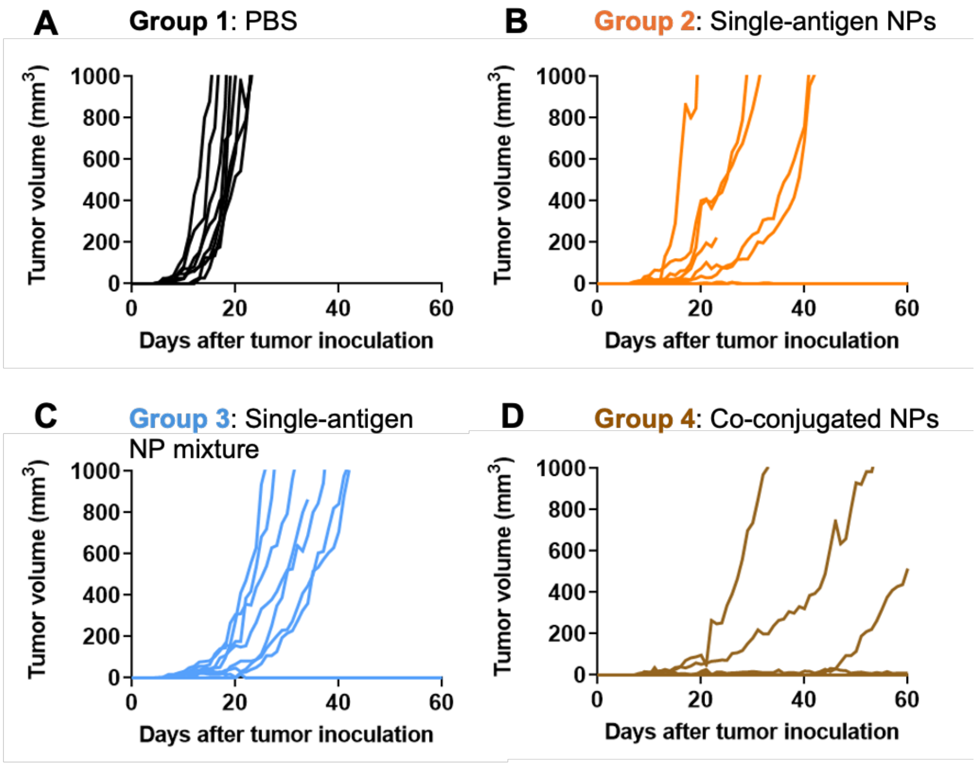

Figure S9. Treatment with E2 NPs decorated with MHC class I and II epitopes delayed colon carcinoma tumor growth. Tumor volumes of individual mice bearing colon carcinoma over time. Tumor growth was monitored over several weeks following initial tumor inoculation on day 0 for mice treated on days 3 and 10 with **(A)** PBS, **(B)** (CT-I)-CpG-E2 + CpG-E2, **(C)** (CT-I)-CpG-E2 + (CT-II)-CpG-E2, and **(D)** (CT-I+II)-CpG-E2 + CpG-E2. Of the four groups, Groups 2 and 3 included treatments with single-antigen NPs, while Groups 4 used NPs co-conjugated with both MHC class I and II epitopes. *N≥7 biological replicates.*

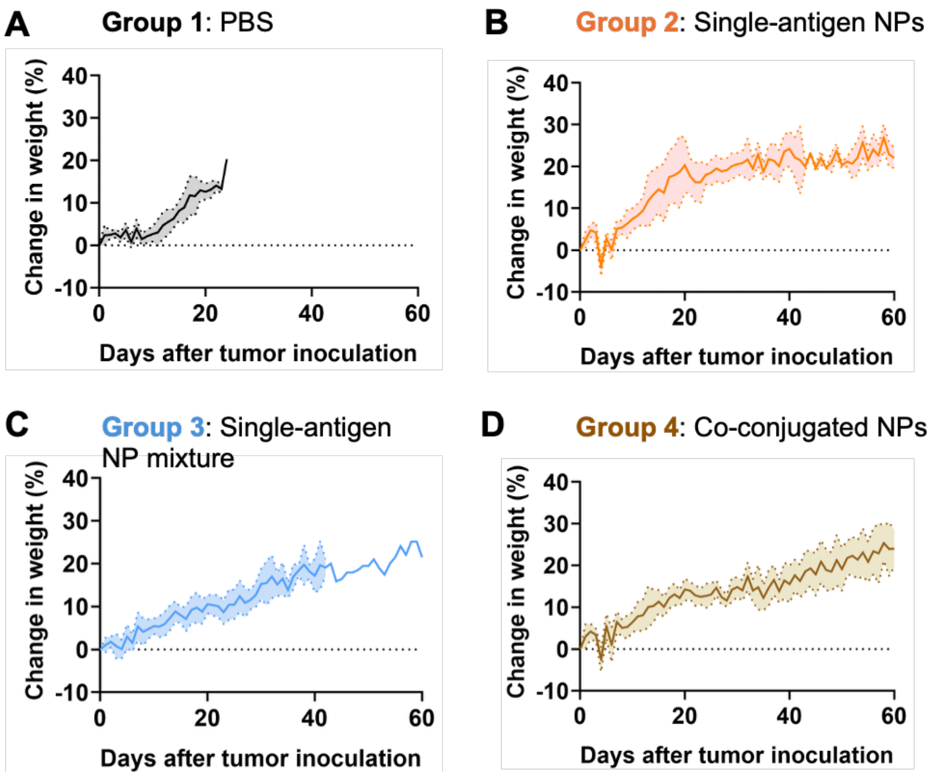

Figure S10. Mouse body weight over time. Shown are the percent changes in mouse body weight over time through day 60 after tumor inoculation, calculated relative to day 0 for each mouse. Treatment groups were: **(A)** PBS, **(B)** (CT-I)-CpG-E2 + CpG-E2, **(C)** (CT-I)-CpG-E2 + (CT-II)-CpG-E2, and **(D)** (CT-I+II)-CpG-E2 + CpG-E2. The dashed horizontal line (y = 0) represents no change in body weight relative to day 0. The shaded area represents the *mean* ± *SD. N≥7 biological replicates.*
